# Disrupting MED8-dependent epigenetic reprogramming augments avapritinib sensitivity in PDGFRA-driven glioma

**DOI:** 10.1186/s13046-026-03736-0

**Published:** 2026-05-19

**Authors:** Han Xie, Zhang Xiong, Ruixiang Huang, Yongfei Dong, Yunlong Wang, Tao Chen, Chunlong Zhong, Yuan Jiang, Dasheng Tian, Erbao Bian

**Affiliations:** 1 Department of Orthopaedics, Institute of Orthopaedics, Research Center for Translational Medicine, The Second Hospital of Anhui Medical University, Hefei, China; 2 School of pharmacy, Anhui Medical University, Hefei, China; 3 Department of Neurosurgery, Shanghai East Hospital, School of Medicine, Tongji University, Shanghai, China; 4 Department of Neurosurgery, The First Affiliated Hospital of Wannan Medical College (Yijishan Hospital of Wannan Medical College), Wuhu, China; 5 Anhui Province Key Laboratory of Medical Physics and Technology, Institute of Health and Medical Technology, Hefei Institutes of Physical Science, Chinese Academy of Sciences, Hefei, China

**Keywords:** Glioma, MED8, Super-enhancer, PDGFRA, Venetoclax

## Abstract

**Background:**

PDGFRA genetic alterations are a well-established oncogenic driver in gliomas. However, targeted monotherapy against PDGFRA such as avapritinib has achieved limited clinical efficacy, and the mechanism underlying avapritinib resistance remains poorly understood.

**Methods:**

Multi-omics analysis of clinical samples identified super-enhancer (SE) complex components. Comprehensive preclinical evaluation was performed using glioma cell lines, glioma stem cells, patient-derived cells, xenografts, and organoids. Mechanistic investigations integrated Cleavage Under Targets and Tagmentation, chromatin immunoprecipitation, co-immunoprecipitation, mass spectrometry, protein fragment complementation, and dual-luciferase reporter assays.

**Results:**

Functional and clinical analyses identified the SE complex component MED8 as significantly upregulated in gliomas and correlated with poor prognosis. MED8 was essential for tumor proliferation and survival both in vitro and in vivo. Mechanistically, MED8 cooperated with CDK7 to bind and activate the SEs of PDGFRA, sustaining high transcriptional output of this oncogene. We repurposed FDA-approved venetoclax as a first-in-class MED8-targeting agent that potently sensitizes to avapritinib, exerting synergistic effects in multiple preclinical models.

**Conclusions:**

This study delineates a novel MED8-SE-PDGFRA epigenetic axis driving resistance. The combination of avapritinib and venetoclax, co-targeting the oncogenic signal and its transcriptional regulator, presents a translatable dual-targeting strategy to improve outcomes in PDGFRA-driven glioma.

**Supplementary Information:**

The online version contains supplementary material available at 10.1186/s13046-026-03736-0.

## Introduction

The malignant progression of glioma is frequently driven by aberrant receptor tyrosine kinase signaling, with platelet-derived growth factor receptor alpha (PDGFRA) amplifications and mutations representing a dominant oncogenic paradigm [1]. These alterations trigger constitutive ligand-independent activation of the PDGFRA tyrosine kinase, leading to the aberrant stimulation of downstream proliferative and survival pathways such as PI3K/AKT and RAS/MAPK, which are fundamental to gliomagenesis and tumor progression [2, 3]. Avapritinib—a potent, selective, brain-penetrant PDGFRA tyrosine kinase inhibitor—exerts meaningful therapeutic efficacy in glioma patients harboring specific activating PDGFRA alterations (i.e., PDGFRA amplification or canonical D842V mutation) [4]. However, inherent drug resistance limits avapritinib monotherapy, creating an urgent unmet clinical need to elucidate resistance mechanisms and develop combination therapies for improved glioma outcomes.

Super-enhancers (SE) complex is a category of molecular complex that binds to and regulates SE activity [5]. It mainly consists of the high density of transcription factors, histone regulators, and SE cofactors [6]. Certain components of the SE complex serves as markers for identifying SEs [7]. Additionally, SE regions can interact with SE complex and RNA polymerase II (RNA Pol II), facilitating the regulation of oncogenes in various tumor types [8]. Notably, cancer cells exhibit heightened sensitivity to the inhibition of SE complex, leading to disrupted transcription of SE-driven oncogenes [9, 10]. Inhibitors targeting SE complex have demonstrated promising anticancer efficacy in various preclinical models [11–13]. However, the regulation of oncogenes by SE complex in glioma and the subsequent impact on the therapeutic sensitivity have largely been uncharacterized.

In this study, we identified MED8 as a critical, prognostically relevant component of the SE complex in gliomas that interacts with CDK7 to regulate PDGFRA transcription. Most importantly, we translated this mechanistic insight into a clinically actionable therapeutic combination by repurposing the FDA-approved drug venetoclax as the first-in-class MED8-targeting agent that sensitizes glioma to avapritinib. Our findings validate a novel, highly effective combination regimen that capitalizes on epigenetic inhibition to augment targeted therapy, offering a readily clinically translatable strategy to improve outcomes for patients with PDGFRA-driven glioma.

## Methods

### Ethics statement

The study was approved by the Research Ethics Committee of The Second Affiliated Hospital of Anhui Medical University, and written informed consent was obtained from all patients. All animal experiments were conducted with the approval of the Animal Research Committee of Anhui Medical University.

### Patient samples

Upon receiving approval from the Research Ethics Committee of the Second Affiliated Hospital of Anhui Medical University, we collected 10 normal brain tissue samples and 30 glioma tissue samples. Informed consent was obtained from all patients prior to sample collection. The information is shown in Supplementary Table S1.

### Human tissue microarray immunohistochemistry

A commercially available high-density tissue microarray comprising 180 glioma cases of varying grades (World Health Organization grades 1–4) and a single control sample was procured from Superchip Biotech (no. HBraG180Su01). Immunohistochemistry (IHC) was performed using anti-MED8 and anti-CDK7 antibodies to detect specific signals. The IHC score was calculated by multiplying the staining intensity by the proportion of positively stained cells.

### Glioma cell lines culture and treatment

All cell lines were tested for mycoplasma contamination and underwent STR cell identification. Procell (Wuhan, China) provided human glioma cell lines such as LN18, H4, T98G, LN229, U251, and SF126. The normal human astrocyte cell line NHA was acquired from Fenghbio (Changsha, China). LN18, U251, H4, NHA, and LN229 cells were cultured in DMEM (Gibco, USA), while T98G and SF126 cells were cultured in MEM (Gibco, USA) with 10% FBS (Gibco, USA) in a 37 °C incubator with a 5% CO_2_ concentration. The transfection method was carried out using Polyplus Transfection Reagent (Polyplus, France) according to the manufacturer’s instructions. Supplementary Table S2 displays all target shRNA sequences from General-Biol, China.

### Glioma Patient-Derived Cells (PDCs)

The glioma PDCs were provided by the Department of Neurosurgery of Shanghai East Hospital [14]. PDCs were cultivated in DMEM/F12 (Gibco, USA) with 10% FBS.

### Glioma Stem Cells (GSCs)

GSCs were cultured in DMEM/F12 supplemented with 2% B27 (Thermo Fisher, USA), 20 ng/ml bFGF, and 20 ng/ml EGF (Thermo Fisher, USA). Cells were seeded in ultra-low attachment culture flasks (Corning, USA) and maintained as tumor spheres.

### Glioma Patient-Derived Organoids (PDOs)

The glioma PDOs were provided by the Department of Neurosurgery of Shanghai East Hospital [14]. The PDOs were cultured in a medium composed of 25% DMEM, 25% DMEM/F12, and 50% Neurobasal, supplemented with 1×Pen-Strep, 1×N2, 1×GlutaMax, 1×B27, 1×NEAA, and 1 × 2-mercaptoethanol. Unlike the previous protocol, we additionally included 20 ng/mL bFGF and 20 ng/mL EGF to enhance organoid growth.

### Immunofluorescence (IF), western blot, and reverse transcription quantitative PCR (RT-qPCR)

IF, western blot, and RT-qPCR were carried out according to established techniques [14]. Supplementary Table S3 contains a list of antibodies utilized. Supplementary Table S4 contains a list of primers used.

### CCK-8 assay

Cell viability was determined using Cell Counting Kit 8 (Biosharp, China). A 96-well plate was used to cultivate 3000 cells, using 100 µL of medium per well. Next, 10 µL of CCK-8 reagent was added to each well and incubated for 2 h. The curve was created using the OD values taken at 450 nm.

### Colony formation assay

After transfection, cells were seeded into 6-well plates at the quantity of 400 cells per well, which allowed single cell colonies to form after 2 weeks of culture. Then, colonies were fixed by 4% paraformaldehyde for 30 min, and 0.5% crystal violet was employed to stain colonies for an additional 30 min. Finally, the number of colonies (more than 50 cells per colony) was counted and photographed utilizing a microscope (Olympus, Japan) and camera.

### Flow cytometry analysis

Before assessing apoptosis, cells were incubated with different compounds following the protocol of the Annexin V-EGFP/PI Double Staining Apoptosis Detection Kit (KeyGEN, China). Cell apoptosis rates were then quantified using flow cytometry (BD Biosciences, USA).

### EdU assay

Cell proliferation was evaluated using the EdU Cell Proliferation Kit (KeyGEN, China) following the manufacturer’s protocol. Fluorescent images were captured using a Nikon microscope.

### Limiting dilution assay

GSCs were dissociated to single cells and then plated in 96-well plates at a density of 10, 20, 30, 40, and 50 cells per well. After 7 days, each well was examined for formation of tumor spheres. Stem cell frequency was measured and analyzed by Extreme Limiting Dilution analysis software (http://bioinf.wehi.edu.au/software/elda).

### Plasmid construction

pcDNA3.1 plasmids encoding MED8 and CDK7 were purchased from Haogebio (Shanghai, China). To generate various truncations of the proteins, the sequence- and ligation-independent cloning method was employed. The PCR products were digested with EcoRI and inserted into the Nhe I/Not I site of a previously digested plasmid containing a C-terminal HA epitope.

### Protein fragment Complementation Assay (PCA)

MED8 and CDK7 were individually fused to complementary N-terminal (Luc1, residues 1-229) or C-terminal (Luc2, residues 230–311) fragments of Renilla luciferase. Each fusion construct was N-terminally tagged with a Flag epitope and cloned into a pcDNA3β expression vector (Miaoling, China). Protein-protein interaction between MED8 and CDK7 was monitored by quantifying the restored Renilla luciferase activity in this Rluc-based protein fragment complementation assay (Rluc-PCA).

### CRISPR/dCas9-KRAB interference

To establish dCas9-KRAB-expressing glioma cells, lentivirus was first generated using the pHAGE-EF1α-dCas9-KRAB plasmid (Addgene, 50919). Transduced cells were selected with puromycin (2 µg/mL; ThermoFisher Scientific) for stable integration. For CRISPR interference (CRISPRi) assays, sgRNAs targeting the SE region of PDGFRA were cloned into a lentiviral sgRNA expression vector and subsequently transduced into the dCas9-KRAB-expressing glioma cells. The sgRNA sequences are listed in Supplementary Table S5.

### Co-immunoprecipitation (Co-IP) assay

Co-IP was utilized to detect the interaction between CDK7 and MED8, and the antibody-protein complex was captured using Protein A/G agarose (Absin, China). The generated complexes were then submitted to western blot analysis using the anti-CDK7 and anti-MED8 antibodies.

### Chromatin immunoprecipitation (ChIP) assay

ChIP analysis was performed according to the manufacturer’s instructions for the SimpleChIP Plus Sonication Chromatin IP Kit (CST, USA). The cells’ chromatin was treated with 2 µg antibodies. RT-qPCR was used to identify immunoprecipitated DNA with specified primers (Supplementary Table S4).

### Dual-luciferase reporter assay

The PDGFRA promoter and SE regions were inserted into the pGL3 promoter and pGL3 enhancer plasmids. The activity of luciferase was evaluated using a Dual-Luciferase Kit (Promega, USA).

### Virtual screening

Based on MED8’s crystal structure (PDB ID: 7EMF), we predicted possible binding sites using a crucial structural domain. Following that, we used a grid-based ligand docking technique with the Grid-based Ligand Docking with Energetics (Glide) program (Schrödinger Suite 2022), which included a cascade docking strategy combining standard precision docking, additional precision docking, and MM/GBSA rescoring. We obtained 10 compounds with high-ranking scores and optimum binding properties from a natural product collection (TargetMol, USA; Supplementary Table S6).

### Cellular Thermal Shift Assay (CETSA)

The cell lysate supernatants were evenly split into two aliquots. One aliquot served as the control group (treated with DMSO), while the other was designated as the drug treatment group and incubated at room temperature for 2 h. Subsequently, both supernatants were further divided into six equal portions and subjected to heat treatment at distinct temperatures for 10 min each. After cooling, loading buffer was added to the supernatants, followed by a 10-minute incubation at 70 °C. Finally, MED8 expression levels were analyzed using western blotting.

### RNA-seq and data analysis

RNA-Seq was performed in sh-MED8 versus sh-con-infected LN18 cells. Novogene (Beijing, China) supplied RNA library creation and sequencing services. Data were processed via the TopHat-Cufflinks pipeline.

### Tumor formation assay in vivo

Beijing HFK Biotechnology provided us with 4-week-old BALB/c nude mice. For orthotopic tumor models, the BALB/c-nude mice were randomly divided into four experimental groups (*n* = 6). The luciferase-expressing cells were injected into the intracranial area of mice. Drug in vivo collaborative experiments were also divided into a vehicle group, venetoclax group, avapritinib group, and venetoclax+avapritinib group. Venetoclax (100 mg/kg, once daily) or avapritinib (30 mg/kg, once daily) were administered by oral gavage. Tumor growth was examined at day 21 using bioluminescence imaging. Each mouse is defined as a biological replicate, with tumor samples harvested from individual mice processed and analyzed separately.

### Drug combination analysis

The synergistic effect of the drug combination was evaluated using the ZIP synergy score and calculated using SynergyFinder 3.0. The ZIP synergy scores were mean ± SEM from three independent biological replicates for all dose-response matrix experiments, with each biological replicate comprising a complete dose matrix performed in technical triplicate. 95% confidence intervals have been calculated for all ZIP scores and are presented in the figures. A ZIP synergy score of (− 10 to 10) indicates an additive effect, and (> 10) indicates synergism.

### Statistical analysis

For comparisons involving more than two groups, one-way ANOVA followed by Tukey’s post hoc test was applied. Survival curves were analyzed using the log-rank test. For RNA-seq and ChIP-seq data, the Benjamini-Hochberg false discovery rate procedure was used to correct for multiple testing. Pairwise comparisons were performed using two-tailed Student’s t-test.

All statistical analyses were performed using GraphPad Prism 10 software on biological replicates. Data represent at least three independent biological replicates, each performed with technical duplicates or triplicates as indicated, with biological replicate defined as cells from a separate culture passage or a separately plated batch on a different day. Data are presented as mean ± SD. Statistical significance was defined as *p < 0.05 and **p < 0.01.

## Results

### SE complex MED8 is frequently overexpressed in glioma and correlated with poor prognosis

**Fig. 1:**
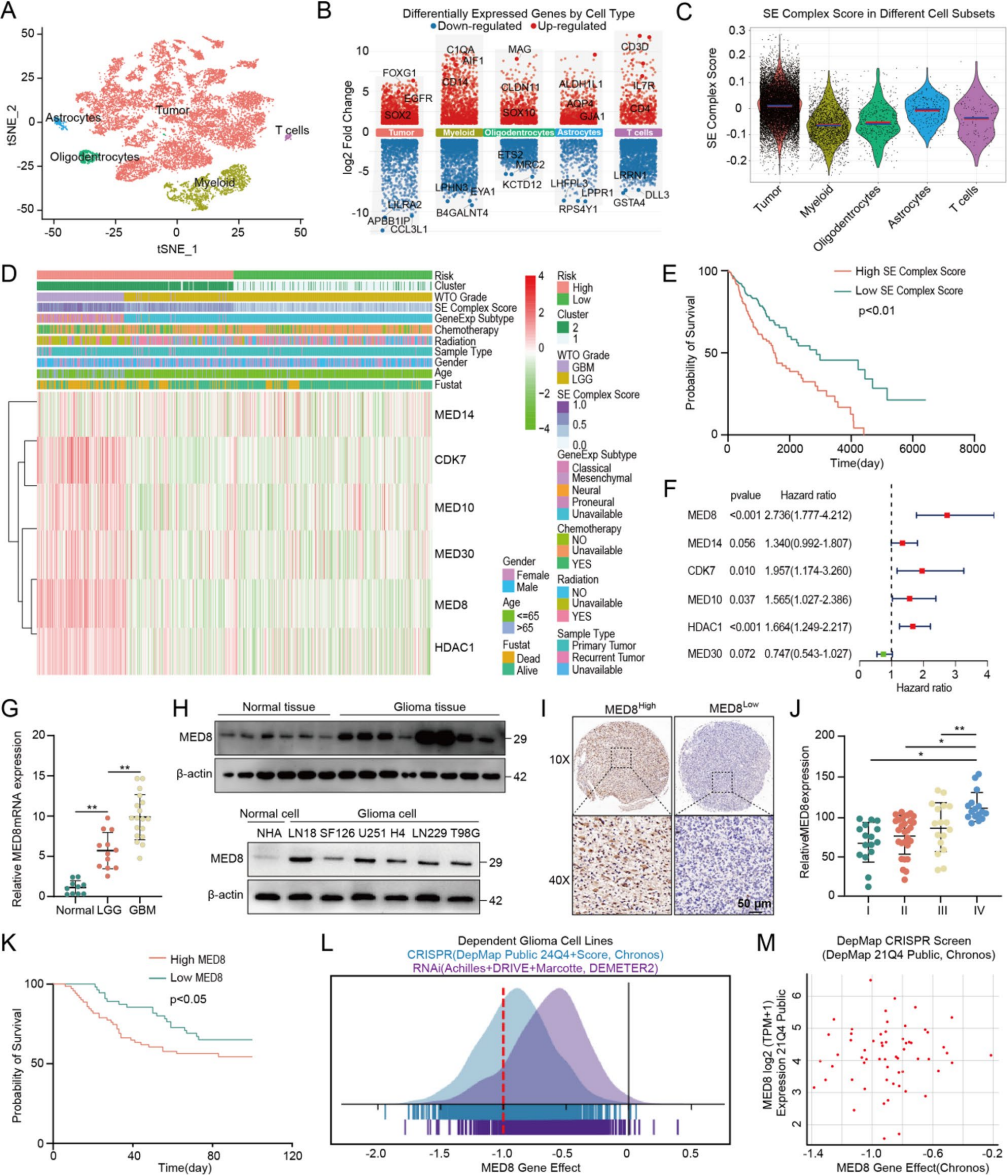
SE complex MED8 is overexpressed in glioma. **A** t-SNE visualization of integrated single-cell RNA-seq data from multiple public GEO datasets, identifying 5 distinct cell lineages. **B** Volcano plot showing significantly upregulated and downregulated genes across the 5 annotated cell types. The plot validates known cell identity markers. **C** Violin plots depicting the distribution of SE complex score for each annotated cell type. **D** The heatmap showed the expression level of 6 SE complexes and the distribution of clinicopathological features in different subgroups. **E** Kaplan-Meier survival analysis on cases with high SE complex score vs. low SE complex score in the TCGA database. **F** Multivariate Cox regression calculated the hazard ratio and regression coefficient of the 6 SE complexes. **G** RT-qPCR analysis of MED8 in 10 normal, 12 LGG tissues, and 18 GBM tissues. **H** Western blot analysis of MED8 in glioma tissues and cell lines. **I** Representative MED8 protein immunohistochemical images were generated on tissue microarrays from a large glioma cohort. **J** Expression levels of MED8 in gliomas of different grades in the cohort. **K** Kaplan-Meier survival analysis on cases with MED8^High^ vs. MED8^Low^ from the cohort. **L**-**M** DepMap CRISPRScreen analysis of MED8 expression and function tolerance in glioma cells. Data represent three independent biological replicates, each performed with technical duplicates or triplicates. Data are presented as mean ± SD. *, *p* < 0.05; **, *p* < 0.01; ns, not significant

Long-range chromatin contacts between SEs and promoters are facilitated by mediator proteins, epigenetic regulators, transcription factors, and co-factors, making SE-related components crucial for robust gene transcription [15, 16]. To investigate the functional role of this regulatory layer in glioma, we first characterized the tumor’s cellular heterogeneity. We integrated multiple publicly available GEO single-cell RNA-seq datasets and performed t-SNE projection, which revealed five distinct cell clusters (Fig. 1A). Differential expression analysis confirmed the identity of each cluster using established cell-type-specific markers, including glioma-associated markers (EGFR, SOX2, FOXG1), myeloid markers (CD14, AIF1, C1QA), oligodendrocyte markers (MAG, SOX10, CLDN11), astrocyte markers (AQP4, ALDH1L1, GJA1), and T-cell markers (CD4, CD3D, IL7R) (Fig. 1B). We then evaluated the SE landscape across these cell types by calculating an SE complex score. Notably, tumor cells exhibited a significantly higher SE complex score compared to all other cell lineages, suggesting an enhanced and dysregulated transcriptional capacity in malignancy (Fig. 1C). To contextualize these findings clinically, we analyzed the expression of 65 SE complex components with prognostic relevance from the TCGA database. Through rigorous screening, we identified 6 key genes and applied multiple consensus clustering methods to stratify patients into two distinct subgroups (Supplementary Fig. S1A-H and Table S7-S8). The resulting ssGSEA scores derived from these genes yielded patient groupings that closely matched conventional risk models, validating the accuracy of our approach (Fig. 1D). Finally, we investigated the clinical relevance of the SE complex score. It was gradually elevated in glioma and demonstrated strong correlations with adverse clinical features, including chemoradiotherapy history, tumor recurrence, and unfavorable patient outcomes (Fig. 1E, Supplementary Fig. S1I).

Given its pronounced expression difference, high hazard ratio, and limited prior research in glioma, MED8 was selected from the candidate genes for further investigation (Fig. 1F). MED8 expression was significantly upregulated in glioma, increased with advancing tumor grade, and was strongly associated with poorer overall survival (Supplementary Fig. S2A-F). Based on the elevated MED8 expression observed in glioma tissues and cell lines (Fig. 1G-H), we selected LN18 and U251 (highest expression) and SF126 (lowest expression) for subsequent experiments. To independently validate the clinical relevance of MED8, we performed immunohistochemical analysis on a tissue microarray from a large glioma cohort. The confirmed association between high tumor grade and shortened survival in this cohort attests to its reliability (Supplementary Fig. S2G-H). MED8 expression was categorized as high or low based on staining intensity and percentage (Fig. 1I). We found that MED8 is strongly expressed in grade II, III, and IV glioma, with an increasing frequency in higher-grade glioma (Fig. 1J). Consequently, patients with MED8^Low^ expression showed significantly improved prognosis compared to those with MED8^High^ expression (Fig. 1K). Finally, we assessed the functional dependency of glioma cells on MED8. Analyses from DepMap and CRISPRScreen databases demonstrated that MED8 was essential for glioma cell growth, as its knockdown suppressed proliferation and glioma cells exhibited a high dependence on MED8 expression (Fig. 1L-M).

### MED8 orchestrates key pro-tumorigenic traits

**Fig. 2:**
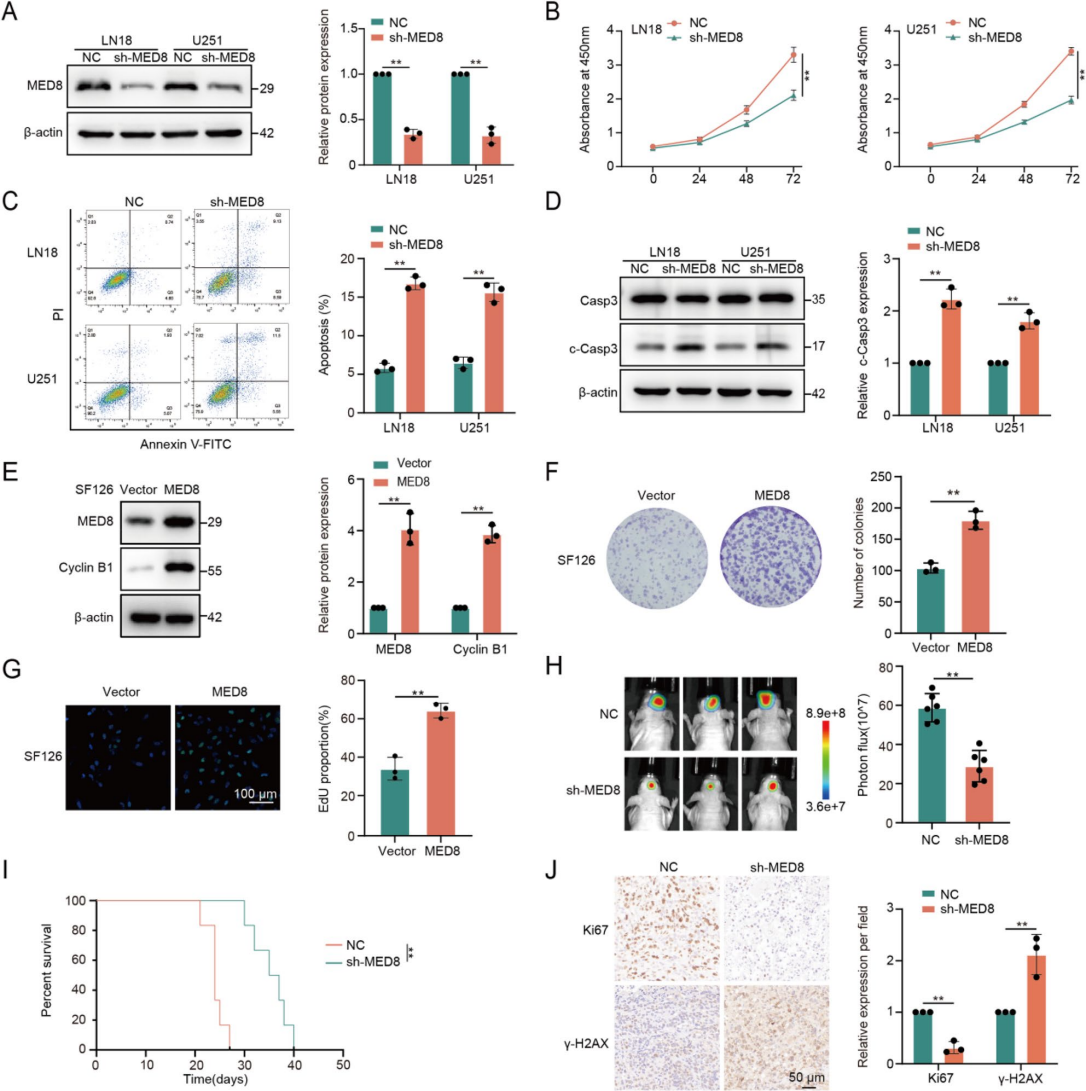
MED8 promotes glioma progression. **A** Western blot analysis of MED8 in NC/sh-MED8 LN18 and U251 cells. **B** CCK8 assays were performed to examine the proliferation. **C** Stained with PI and annexin V-FITC for apoptotic analysis. Gate set to distinguish early (Q3: Annexin V+/PI−) and late (Q2: Annexin V+/PI+) apoptotic cells. Q1: Necrosis cells; Q4: Viable cells. **D** Western blot analysis of cleaved caspase-3 in NC/sh-MED8 LN18 and U251 cells. **E** Western blot analysis of MED8 and Cyclin B1 in SF126 cells transfected with MED8 plasmids. **F** Colony formation assay of SF126 cells transfected with MED8 plasmids. **G** Confocal IF showed the percentage of EdU-positive cells transfected with MED8 plasmids. **H** Bioluminescent imaging and quantification of intracranial tumor growth of mice derived from the luciferase-expressing U251 cells transduced with sh-MED8 (*n* = 6). **I** Kaplan-Meier survival curves for nude mice. **J** Representative Ki-67 and γ-H2AX IHC staining in mice xenografts. Data represent three independent biological replicates, each performed with technical duplicates or triplicates. Data are presented as mean ± SD. *, *p* < 0.05; **, *p* < 0.01; ns, not significant

To elucidate the functional significance of MED8 in glioma, we performed loss-of-function and gain-of-function analyses. We began by knocking down MED8 expression in two glioma cell lines (LN18 and U251) exhibiting high endogenous MED8 levels (Fig. 2A). MED8 knockdown led to a pronounced suppression of cellular proliferation (Fig. 2B). Furthermore, flow cytometry revealed a significant increase in apoptosis upon MED8 depletion, which was corroborated by elevated levels of cleaved caspase-3, indicating enhanced activation of apoptosis (Fig. 2C-D). Extending our investigation to GSCs, we established several GSC lines cultured in serum-free neural stem cell medium. A line exhibiting notably high MED8 expression was selected for further analysis (Supplementary Fig. S3A). Consistent with the pro-tumorigenic functions observed in conventional glioma cell lines, MED8 knockdown in GSCs significantly suppressed cell proliferation and promoted apoptosis (Supplementary Fig. S3B-E). To determine whether MED8 overexpression alone is sufficient to drive oncogenic phenotypes, we ectopically expressed MED8 in SF126 cells, which display low basal MED8 expression (Fig. 2E). Notably, MED8 overexpression significantly enhanced clonogenic ability and upregulated Cyclin B1, a key regulator of cell proliferation (Fig. 2E-F). Moreover, EdU staining provided confirmation that the percentage of EdU-positive cells was increased following MED8 overexpression, which was consistent with clonogenic assays (Fig. 2G). We next extended our investigation to an in vivo setting by establishing a xenograft model using MED8-knockdown cells. Consistent with the in vitro data, bioluminescent imaging demonstrated that MED8 depletion markedly suppressed tumor growth and significantly prolonged survival in mice (Fig. 2H-I). Histological examination of xenograft tumors further indicated that MED8 knockdown resulted in reduced Ki-67 staining, indicative of impaired proliferation, along with increased γ-H2AX signals, reflecting elevated DNA damage (Fig. 2J). Collectively, these integrated in vitro and in vivo findings establish MED8, a core component of the super-enhancer complex, as a critical driver of glioma progression.

### PDGFRA is a crucial target of MED8

**Fig. 3:**
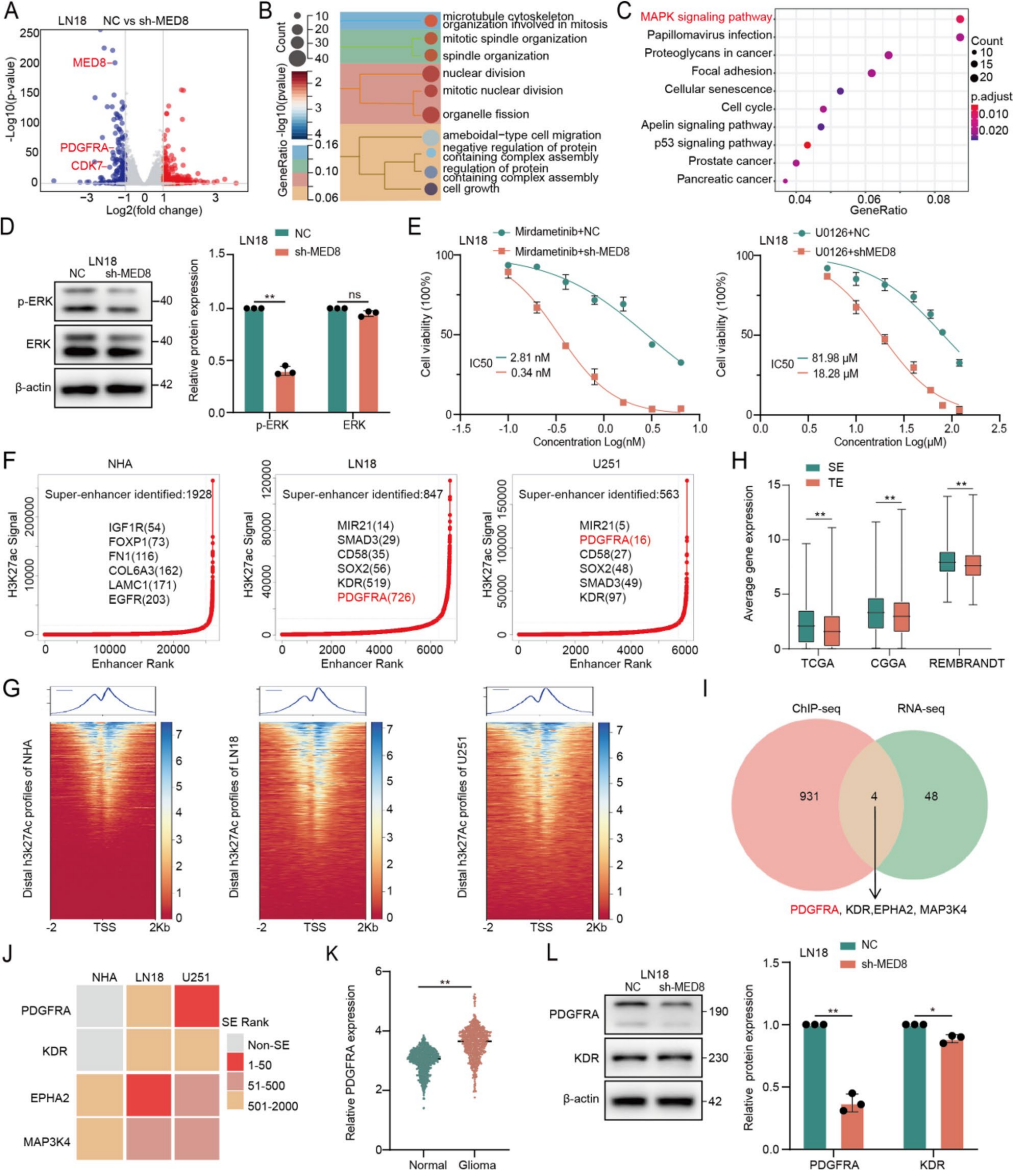
PDGFRA is a crucial target of MED8. **A** Volcano plot of differential gene expression between NC and sh-MED8 LN18 cells (blue, downregulated; red, upregulated). **B**-**C** The gene ontology (GO) and Kyoto Encyclopedia of Genes and Genomes (KEGG) analysis of the differential genes. **D**Western blot analysis of p-ERK and ERK in NC/sh-MED8 LN18 cells. **E** CCK8 assays were performed to examine the effect of MED8 knockdown on the IC50 of ERK kinase inhibitors (U0126 and mirdametinib) in LN18 cells. **F** Enhancer regions of the normal astrocyte cells (NHA) and glioma cells (LN18 and U251). **G** Dynamic H3K27ac peaks in NHA, LN18, and U251 cells. **H** Expression of SE and TE genes in multiple databases. **I** The Venn diagram showed that between the ChIP-seq and RNA-seq, only four genes were unique. **J** A rectangular chart showed the rankings of four SE genes. **K** Expression level of PDGFRA in the TCGA database. **L** Western blot analysis of PDGFRA and KDR in NC/sh-MED8 LN18 cells. Data represent three independent biological replicates, each performed with technical duplicates or triplicates. Data are presented as mean ± SD. *, *p* < 0.05; **, *p* < 0.01; ns, not significant

To systematically identify the downstream pathways regulated by MED8, we performed RNA sequencing following MED8 knockdown. This analysis identified 650 differentially expressed genes (249 downregulated and 401 upregulated) with a fold change greater than two (Fig. 3A). Gene Ontology enrichment analysis indicated that the most significantly affected biological processes were organelle fission and mitotic nuclear division, suggesting a role for MED8 in cell cycle progression (Fig. 3B). To pinpoint the specific signaling pathways involved, we performed KEGG pathway analysis. This highlighted the MAPK signaling pathway as a top candidate, which we selected for further validation given its established role in glioma pathogenesis (Fig. 3C). We next focused on the MAPK pathway for mechanistic investigation. Analysis of our RNA-seq data confirmed that the expression of a core set of MAPK signature genes was consistently downregulated upon MED8 knockdown (Supplementary Fig. S4A). In addition, the MAPK signaling pathway includes three classical signaling pathways, namely extracellular regulated protein kinases (ERK), p38 MAP kinase (p38), and c-Jun N-terminal kinase (JNK). Specifically, the ERK pathway is primarily engaged in cell proliferation and differentiation [17]. We found that ERK phosphorylation was significantly reduced in MED8 knockdown glioma cells, whereas JNK and p38 remained unchanged (Fig. 3D, Supplementary Fig. S4B-C). Consistent with our findings in conventional glioma cell lines, MED8 knockdown in GSCs led to a marked downregulation of PDGFRA mRNA levels, accompanied by reduced ERK phosphorylation, further confirming the MED8-PDGFRA-ERK axis in this clinically relevant model (Supplementary Fig. S4D-E). To further establish the functional connection between MED8 and ERK signaling, we assessed whether MED8 depletion sensitizes glioma cells to ERK pathway inhibition. Consistent with our hypothesis, MED8 knockdown significantly reduced the half-maximal inhibitory concentration (IC50) of two distinct ERK kinase inhibitors, U0126 and mirdametinib (Fig. 3E, Supplementary Fig. S4F). Collectively, these data pinpoint the MAPK/ERK axis as a key downstream signaling pathway through which MED8 promotes glioma progression.

To investigate the role of SE alterations in glioma pathogenesis, we compared the SE landscapes of normal human astrocytes (NHA) with two glioma cell lines. SEs were defined by stitching constituent enhancers within 12.5 kb and identifying inflection points in the H3K27ac ChIP-seq signal (Fig. 3F-G). This analysis identified 739 glioma-acquired SE-associated genes, with 228 shared by both glioma cell lines (Supplementary Fig. S5A). These shared SE-associated genes were significantly upregulated in glioma tissues compared to normal brain samples (Supplementary Fig. S5B) and consistently exhibited higher expression levels than genes linked to typical enhancers (TEs) across multiple independent datasets (Fig. 3H). Functional enrichment analysis further demonstrated their involvement in cancer-critical biological processes and key signaling pathways driving glioma progression (Supplementary Fig. S5C-D), suggesting that glioma cells exploit SE-mediated transcriptional amplification of oncogenes to sustain malignancy.

To identify key SE-associated transcripts functionally linked to MED8 in glioma, we performed an integrative analysis of RNA-seq and H3K27ac ChIP-seq data following MED8 knockdown. We applied a multi-step filtering strategy to pinpoint high-confidence candidate genes, requiring them to be: (i) components of the MAPK signaling pathway that were downregulated upon MED8 inhibition; (ii) associated with top-ranked SEs (top 50); and (iii) highly expressed in glioma. This stringent approach yielded four candidate genes: PDGFRA, KDR, EPHA2, and MAP3K4 (Fig. 3I). We next validated these candidates against independent genomic features. Only PDGFRA and KDR were confirmed to be associated with glioma-acquired SEs and to maintain high expression levels in glioma tissues (Fig. 3J-K, Supplementary Fig. S5E). Between these two, PDGFRA expression was the most profoundly suppressed by MED8 knockdown (Fig. 3L, Supplementary Fig. S5F). Given its status as a core MED8 target embedded within the highly active MAPK pathway, PDGFRA was selected for further mechanistic investigation.

### MED8 regulates the SE-associated oncogene PDGFRA

**Fig. 4:**
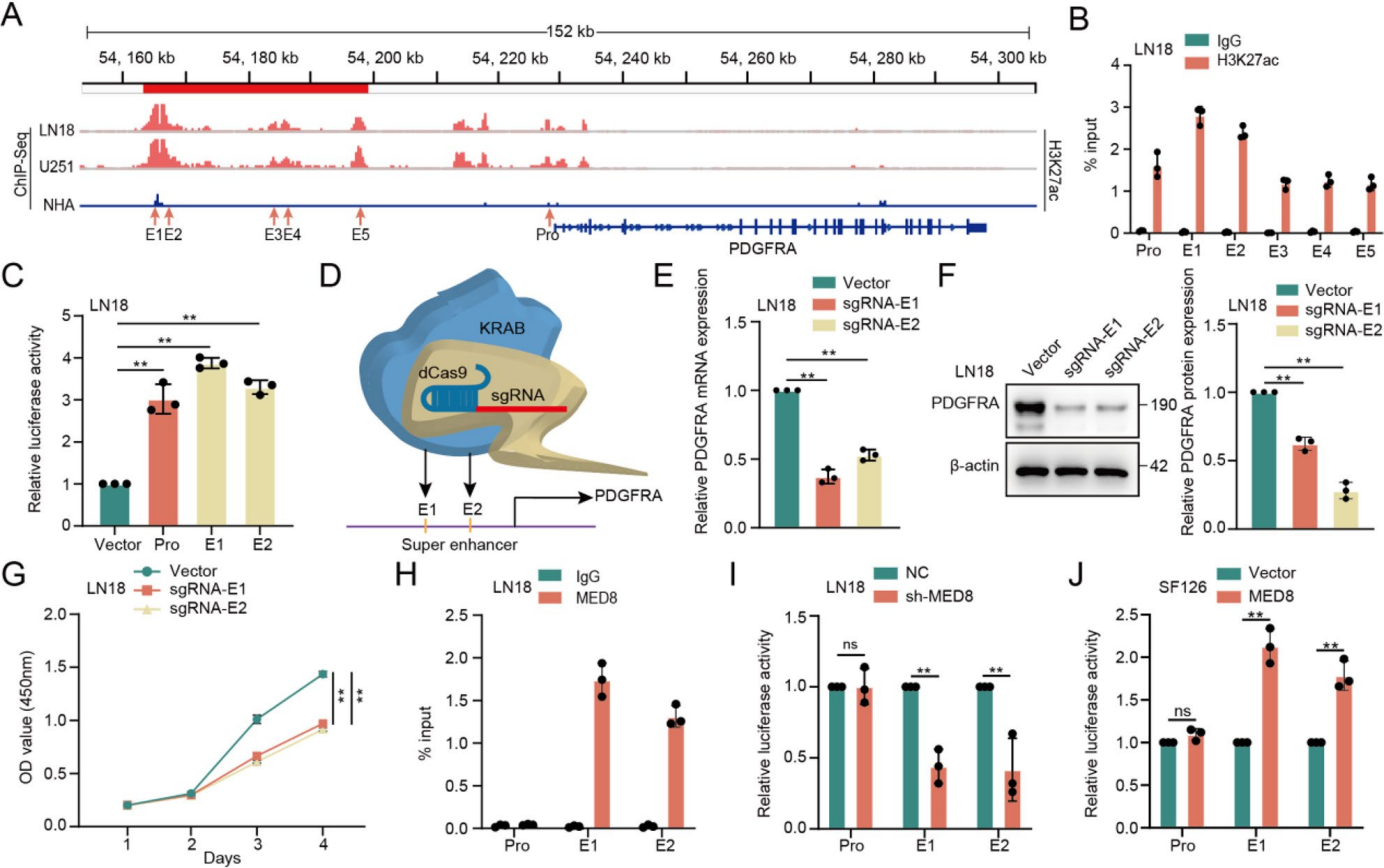
MED8 regulates the SE-associated PDGFRA. **A** Genome browser image at ChIP-seq profiles for H3K27ac at PDGFRA locus in glioma cells and astrocytes. **B** ChIP-qPCR was performed to quantify the enrichment of H3K27ac at the promoter and enhancer regions of PDGFRA in LN18 cells. **C** Luciferase reporter assays measured promoter and enhancer (E1 and E2) activity of PDGFRA in LN18 cells. **D** sgRNAs designed to target the PDGFRA SE critical region using the dCas9-KRAB. **E**-**F** Blockade of PDGFRA SE critical regions (E1 and E2) by sgRNA reduced the expression of PDGFRA in LN18 cells. **G** Blockade of PDGFRA SE critical regions (E1 and E2) by sgRNA reduced the proliferation of LN18 cells. **H** The enrichment of MED8 on the promoter and enhancer of PDGFRA was analyzed with ChIP assays in LN18 cells. **I** Luciferase reporter assays measured promoter and enhancer activity of PDGFRA in NC/sh-MED8 LN18 cells. **J** Luciferase reporter assays measured promoter and enhancer activity of PDGFRA in SF126 cells transfected with MED8 plasmids. Data represent three independent biological replicates, each performed with technical duplicates or triplicates. Data are presented as mean ± SD. *, *p* < 0.05; **, *p* < 0.01; ns, not significant

Our findings showed that glioma cells can acquire SEs around oncogenes such as PDGFRA. Analysis of H3K27ac signals revealed a specific enrichment in the upstream regulatory region of PDGFRA in glioma cells, which was absent in normal human astrocytes, indicating the de novo epigenetic acquisition of this SE in malignancy (Fig. 4A). ChIP-qPCR analysis confirmed significant H3K27ac enrichment at both the SE and promoter regions of PDGFRA (Fig. 4B, Supplementary Fig. S6A). Luciferase reporter assays demonstrated that the promoter and two key SE components (E1, E2) of PDGFRA possessed transcriptional activity in glioma cells (Fig. 4C, Supplementary Fig. S6B). Consistent with findings in conventional glioma cell lines, ChIP-qPCR and luciferase reporter assays confirmed specific enrichment of MED8 at the PDGFRA SE region in GSCs (Supplementary Fig. S6C-D). To functionally interrogate the necessity of this SE, we employed a CRISPR interference system [18, 19]. Specifically, sgRNAs were created to target numerous sites in the SE region, facilitating the recruitment of dCas9-KRAB to disrupt the SE-promoter interaction (Fig. 4D). Notably, targeting two distinct SE sites significantly suppressed PDGFRA expression and impaired glioma cell viability, confirming the SE’s critical role in sustaining PDGFRA transcription and glioma cell survival (Fig. 4E-G, Supplementary Fig. S6E-G).

We then sought to determine how MED8 regulates this circuit. Notably, ChIP analysis demonstrated that MED8 binds specifically to the SE of PDGFRA in glioma cells, with no significant enrichment at its promoter (Fig. 4H, Supplementary Fig. S6H). Corroborating this binding specificity, luciferase reporter assays showed that the transcriptional activity driven by the SE of PDGFRA was significantly modulated by either knockdown or overexpression of MED8 (Fig. 4I-J, Supplementary Fig. 6I). We next examined whether MED8 is required for Pol II recruitment to the PDGFRA promoter. As shown in Supplementary Fig. S6J-K, MED8 knockdown significantly reduced Pol II enrichment at the PDGFRA promoter in GSCs, indicating impaired Pol II recruitment or transcriptional initiation. Collectively, these findings demonstrate that MED8 binds to the PDGFRA SE and is required for SE-dependent Pol II recruitment, establishing MED8 as a critical mediator of enhancer-promoter communication that sustains PDGFRA transcription in glioma.

### MED8 and CDK7 cooperatively activate the SE of PDGFRA

**Fig. 5:**
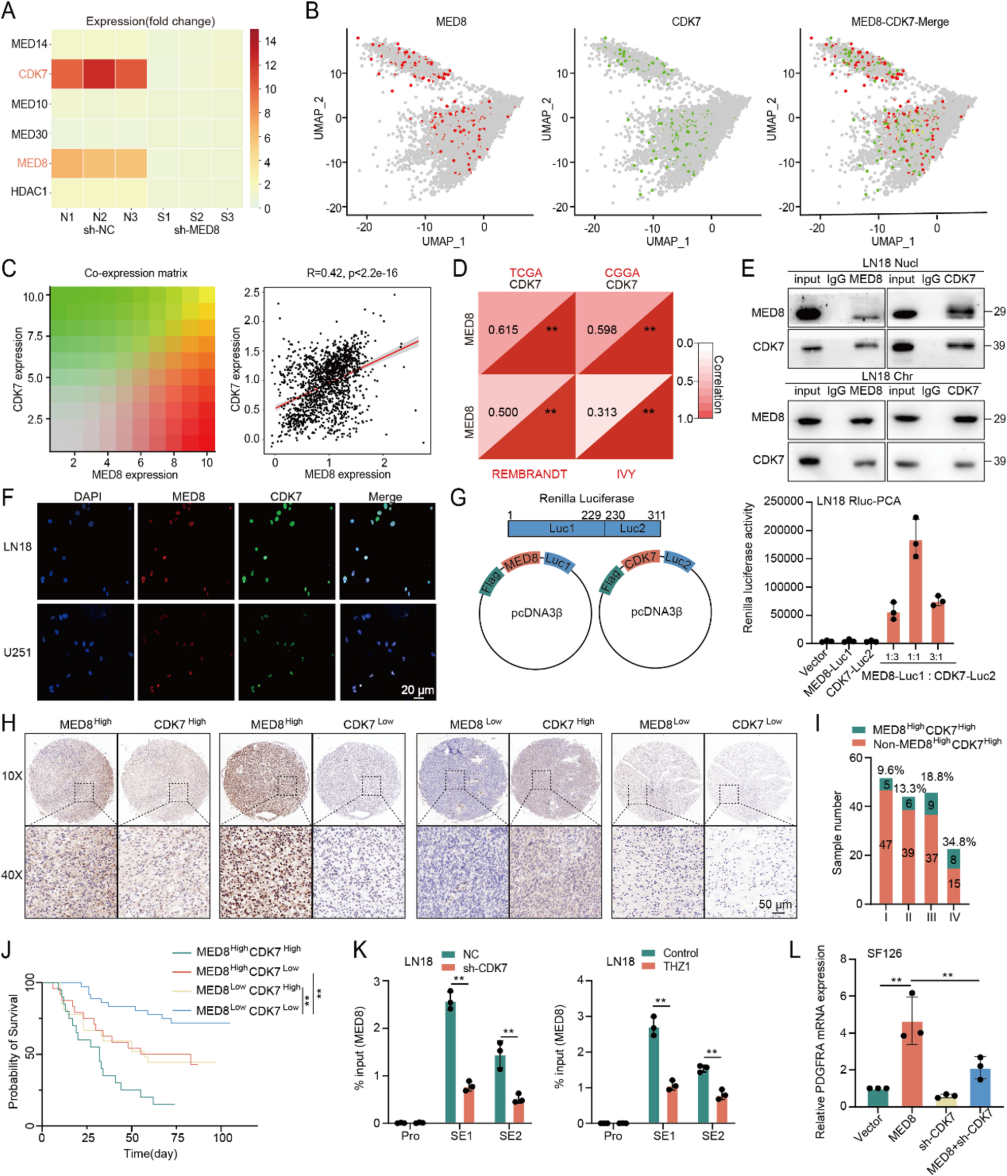
MED8 interacts with CDK7 to regulate the SE of PDGFRA. **A** A rectangular chart showed the changes in the expression of 6 SE complex components in RNA-seq. **B** UMAP plots showing the co-expression of MED8 and CDK7 across multiple public GEO datasets. Red/green dots represent glioma cells expressing MED8 + only/CDK7 + only, respectively, and yellow dots indicate glioma cells co-expressing MED8 and CDK7 only. **C** The co-expression matrix across glioma cells with different expressions of MED8 and CDK7. The correlation (Pearson) between MED8 and CDK7 across MED8 + glioma cells. **D** Correlation between MED8 and CDK7 expression in the TCGA, CGGA, REMBRANDT, and IVY databases. The number represented the correlation coefficient (Cor). **E** Reciprocal Co-IP analysis demonstrated the interaction between MED8 and CDK7 in the LN18 nucleus and chromatin. **F** The co-localization of MED8 with CDK7 was analyzed in LN18 and U251 cells by the IF experiment. **G** Characterization of the MED8-CDK7 Rluc-PCA platform using measurement of Renilla luciferase activity. Transfection of pcDNA3β vector only and individual MED8-Luc1 and CDK7-Luc2 was used as negative controls. **H** Representative MED8 and CDK7 protein immunohistochemical images were generated on tissue microarrays from a large glioma cohort. **I** Case count with different protein expression patterns from the cohort according to tumor grades. **J** Kaplan-Meier survival analysis on different group cases from the cohort. **K** After sh-CDK7 or THZ1 treatment of LN18 cells, ChIP assays were performed to analyze the enrichment of MED8 on the promoter and enhancer of PDGFRA. (L) RT-qPCR was performed to observe the PDGFRA expression in SF126 cells transfected with MED8 plasmid or/and sh-CDK7. Data represent three independent biological replicates, each performed with technical duplicates or triplicates. Data are presented as mean ± SD. *, *p* < 0.05; **, *p* < 0.01; ns, not significant

To identify potential MED8 co-regulators within the SE complex, we reanalyzed MED8 knockdown transcriptome data and identified CDK7 as the most significantly downregulated gene (Figs. 3A and 5A). CDK7, a cyclin-dependent kinase, is a subunit of transcription factor IIH (TFIIH), playing a critical role in the initiation of transcription by phosphorylating RNA Pol II [20]. To investigate the association between CDK7 and MED8 transcripts in glioma, we explored publicly available scRNA-seq databases. Co-expression analysis revealed that MED8-high glioma cells largely overlap with CDK7-high glioma cells, showing a significant positive correlation between MED8 and CDK7 expression at the single-cell level (Fig. 5B-C). Furthermore, this significant positive correlation was consistently observed across bulk transcriptome datasets (Fig. 5D).

To investigate the potential mutual regulation between MED8 and CDK7, we performed a series of interaction assays. First, the interaction between MED8 and CDK7 was measured using Co-IP. Additionally, we innovatively demonstrated the interaction between MED8 and CDK7 at the chromatin level (Fig. 5E, Supplementary Fig. S7A). Next, we determined through an IF dual-labeling experiment that CDK7 and MED8 showed a significant co-localization phenomenon in the cell nucleus (Fig. 5F). We next employed a Renilla luciferase protein fragment complementation assay (Rluc-PCA) to assess the interaction between MED8 and CDK7. As shown in Fig. 5G, a significant bioluminescence signal was detected exclusively when MED8-Luc1 and CDK7-Luc2 were coexpressed. We then evaluated the clinical relevance of this interaction in a glioma tissue cohort (Fig. 5H, Supplementary Fig. S7B). Cases with MED8^High^/CDK7^High^ showed an increased percentage with tumor progression, whereas those with MED8^Low^/CDK7^Low^ had a significantly better prognosis than those with MED8^High^/CDK7^High^ (Fig. 5I-J).

Finally, we investigated the functional interplay between MED8 and CDK7 at the SE of PDGFRA. ChIP-qPCR analysis demonstrated that CDK7 knockdown significantly reduced MED8 binding at the SE of PDGFRA (Fig. 5K, Supplementary Fig. S7C-D), indicating that CDK7 facilitates the recruitment or stabilization of MED8 at this locus. In a functional rescue experiment, CDK7 knockdown partially but significantly attenuated the upregulation of PDGFRA expression induced by MED8 overexpression (Fig. 5L). Collectively, these data demonstrated that CDK7 contributes to the epigenetic activation of PDGFRA by mediating the recruitment or stabilization of MED8 at its SE.

### Synergistic effects of MED8 inhibitor venetoclax and avapritinib

**Fig. 6:**
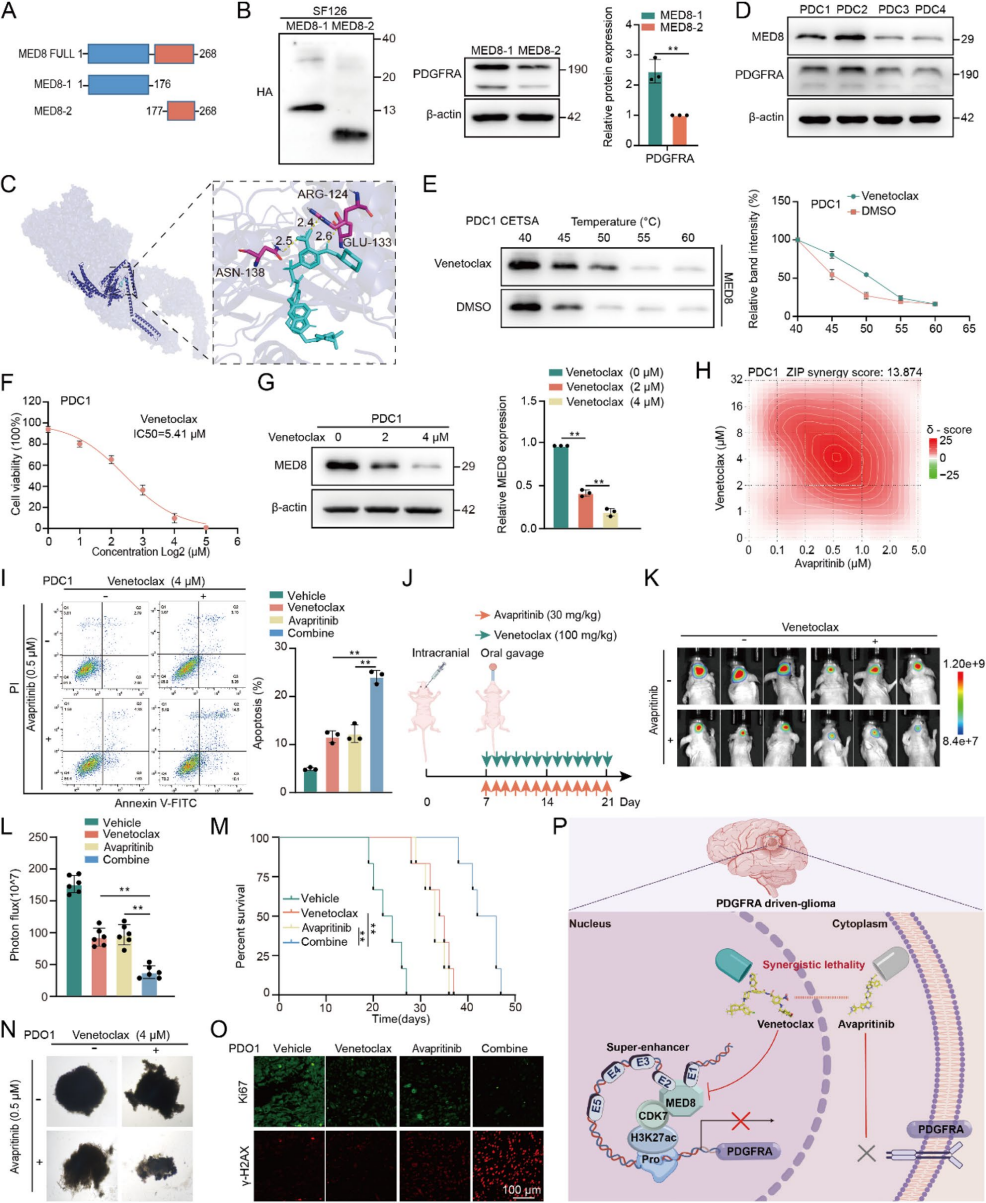
Synergistic effects of MED8 inhibitor venetoclax and avapritinib. **A** Constructors were prepared by adding HA tags to truncated plasmids. **B** Western blot analysis of HA and PDGFRA in SF126 cells transfected with two truncated MED8 plasmids. **C** Molecular simulations using HDOCK were performed to explore the potential interactions between MED8 and venetoclax. **D** Western blot analysis of MED8 and PDGFRA in PDCs. **E** PDC1 were treated with DMSO and venetoclax (4 µM) for 2 h and heated at the indicated temperatures and then lysed for western blot analysis to detect the expression of MED8 protein. CETSA curves indicate relative MED8 band intensity. **F** PDC1 were treated with 1, 2, 4, 8, 16, and 32 µM doses of venetoclax for 48 h. The cell viability was assessed by CCK-8 analysis. **G** Western blot analysis of MED8 in PDC1 treated with 2 and 4 µM doses of venetoclax. **H** Synergy efficacy was observed using the combination of venetoclax and avapritinib that was assessed by the ZIP synergy score. **I** PDC1 were treated with venetoclax and avapritinib for 48 h and harvested for apoptotic analysis. **J** Schematic diagram of venetoclax and avapritinib synergistic therapy after intracranial tumor formation in nude mice. **K**-**L** Bioluminescent image showing the tumor size of mice implanted with luciferase-labeled PDC1. The quantification histogram represents the bioluminescent flux. The mice were selected and randomized into four groups (*n* = 6) and were initiated treatment with venetoclax, avapritinib, venetoclax+avapritinib, or drug vehicle. **M** Kaplan-Meier survival curves for mice. **N** Representative images of PDOs after indicated drug treatments for 5 days: the morphology and size of the organoids were observed and analyzed. **O** IF of Ki-67 and γ-H2AX in PDOs after indicated drug treatments for 5 days. **P** Proposed model illustrating that targeting MED8-mediated epigenetic reprogramming boosts avapritinib sensitivity in PDGFRA-driven glioma. Data represent three independent biological replicates, each performed with technical duplicates or triplicates. Data are presented as mean ± SD. *, *p* < 0.05; **, *p* < 0.01; ns, not significant

While tyrosine kinase inhibitors like avapritinib suppress PDGFRA signaling, they fail to counteract the high transcriptional output of the PDGFRA gene—a key mechanism maintaining oncogenic protein levels and limiting therapeutic durability [4]. Given our discovery that MED8 directly governs PDGFRA transcription, we hypothesized that targeting MED8 could overcome this limitation. To identify a MED8-targeting agent, we first defined its functional domain through structural analysis. Overexpression of this domain (MED8-1) confirmed its role in upregulating PDGFRA (Fig. 6A-B). Virtual screening of FDA-approved compounds against the MED8-1 structure identified venetoclax as a top candidate with favorable predicted binding affinity (Fig. 6C, Supplementary Table S6).

PDCs were utilized for in vitro drug testing because they better preserve the genetic and phenotypic heterogeneity of the original patient tumors compared to traditional, long-term cultured glioma cell lines [21, 22]. Therefore, we selected MED8-high PDCs for subsequent experiments (Fig. 6D). The CETSA revealed that venetoclax significantly increased the thermal stability of MED8 relative to the control group, suggesting a direct interaction between venetoclax and intracellular MED8 (Fig. 6E, Supplementary Fig. S8A). Next, we focused our validation on venetoclax, which has shown significant efficacy in treating certain types of leukemia [23]. Preclinical studies have shown that the combination of venetoclax and radiotherapy has clinical potential for the treatment of diffuse midline gliomas [24]. Therefore, it has become a highly promising candidate drug for reuse in the treatment of glioma. Indeed, treatment with venetoclax exhibited potent anti-proliferative effects (Fig. 6F, Supplementary Fig. S8B-C) and concurrently reduced MED8 protein levels (Fig. 6G, Supplementary Fig. S8D), corroborating its on-target activity. To further establish that venetoclax sensitivity is specifically mediated by MED8, we performed MED8 knockdown and assessed drug responses. As shown in Supplementary Fig. S8E-F, MED8 knockdown significantly increased the IC50 of venetoclax. In contrast, the IC50 of avapritinib was not significantly affected by MED8 knockdown. The preservation of avapritinib sensitivity alongside impaired venetoclax sensitivity thus supports a pathway-specific role for MED8 in mediating venetoclax activity.

To evaluate the therapeutic potential of simultaneously targeting PDGFRA signaling and its transcriptional regulator MED8, we investigated the combination of avapritinib and venetoclax. Using a dose-response matrix analyzed by the ZIP model, we observed robust synergistic effects in PDCs, with high synergy scores of 13.87 and 12.72, respectively (Fig. 6H, Supplementary Fig. S9A). Critically, this synergy was demonstrated to be MED8-dependent, as it was drastically attenuated upon MED8 knockdown (Supplementary Fig. S9B), while MED8 overexpression significantly enhanced the synergistic effect (Supplementary Fig. S9C). To exclude the possibility that the synergy is mediated through BCL-2—the canonical target of venetoclax—rather than through MED8 [25], we performed BCL-2 knockdown in PDCs, which did not attenuate the synergistic effect (Supplementary Fig. S9D-E). Collectively, these data demonstrate that the venetoclax-avapritinib synergy is MED8-dependent and not attributable to BCL-2 inhibition. At an optimized venetoclax concentration of 4 µM, the combination regimen induced a significantly higher rate of apoptosis and a more profound suppression of colony formation compared to either single agent (Fig. 6I, Supplementary Fig. S9F-I).

Building on these compelling in vitro results, we advanced to in vivo validation in PDC xenograft model. The combination of venetoclax with avapritinib demonstrated markedly superior efficacy over all monotherapy groups. Bioluminescence imaging confirmed profound suppression of tumor growth, which translated into a significant extension of median survival (Fig. 6K-M). Consistent with the improved therapeutic efficacy, IHC staining confirmed that the combination group exhibited a marked reduction in proliferation (Ki-67 positivity) and a concomitant elevation in DNA damage (γ-H2AX foci) relative to avapritinib monotherapy (Supplementary Fig. S10A). To establish a pharmacodynamic linkage between the proposed transcriptional mechanism and in vivo response, we analyzed intracranial tumor tissues from treated mice. The results revealed that venetoclax monotherapy significantly reduced MED8 protein expression and reduced PDGFRA mRNA levels (Supplementary Fig. S10B-C). In contrast, the combination achieved significantly greater reduction in p-ERK signaling than either monotherapy alone (Supplementary Fig. S10B). These findings support a dual-layer inhibition model in which venetoclax suppresses MED8-dependent PDGFRA transcription while avapritinib inhibits residual PDGFRA kinase activity, together achieving synergistic efficacy.

PDOs have emerged as a powerful preclinical model that closely recapitulates patient tumors for therapeutic response prediction [26, 27]. In a PDGFRA-driven PDO model, the combination therapy induced profound morphological disruption and a significant reduction in organoid size, effects that were substantially more pronounced than those of either monotherapy (Fig. 6N, Supplementary Fig. S10D). This phenotypic regression was corroborated by a synergistic decrease in Ki-67 and an increase in γ-H2AX foci (Fig. 6O). Collectively, these findings establish the synergistic antitumor activity of the venetoclax and avapritinib combination in PDGFRA-driven glioma.

## Discussion

Glioma represents the most common malignant brain tumor, with amplification or mutation of PDGFRA serving as a pivotal molecular event driving tumorigenesis in a significant subset [28, 29]. Although targeted agents such as avapritinib have been developed, their efficacy as monotherapies in glioma remains limited. This limitation arises because avapritinib, while potently inhibiting downstream PDGFRA signaling, does not address the persistent high-level oncoprotein output driven by epigenetic PDGFRA transcription [4]. In this study, we found that MED8, an SE-associated complex component, is markedly overexpressed in glioma. MED8 interacts with CDK7 and directly binds to the PDGFRA SE to sustain PDGFRA transcription. Importantly, the novel MED8 inhibitor venetoclax, when combined with avapritinib, synergistically suppresses PDGFRA-driven glioma growth in preclinical models (Fig. 6P). This strategy that co-targets oncogene transcription and signaling via genetic-epigenetic dual regulation provides a promising therapeutic option to improve PDGFRA-driven glioma patient outcomes.

The SE complex is an assembly of various proteins, transcription factors, cofactors, and chromatin regulators that converge at SE regions [14]. These components play a crucial role not only in maintaining normal cellular functions but also in contributing to pathological conditions, such as inflammatory diseases and cancer [30, 31]. In cancer, the SE complex regulates the high-level expression of oncogenes by binding to SE regions associated with these genes [32, 33]. Previous studies from our group demonstrated that SE complex components like MED1 and CDK7 contribute to glioma malignant progression [34]; however, the roles of other core components and their cooperative functions in glioma evolution remain incompletely understood. In this study, using integrated bioinformatic classification approaches coupled with experimental validation, we identified MED8, a key component of the SE complex, as critically involved in glioma progression. MED8 performs an integral function in the transcriptional regulation of all eukaryotic organisms, and alterations in its function or composition may have significant pathological consequences, contributing to various diseases, including cancer [35]. However, the specific mechanism and function of MED8 in glioma had not been elucidated. Our findings reveal that MED8 is significantly upregulated in a large glioma cohort and is strongly associated with poor patient prognosis. Further functional findings, both in vitro and in vivo, provided compelling evidence that MED8 drives the progression of glioma. A future study is needed to determine MED8’s precise involvement in the tumor microenvironment and to explore the molecular basis of its heterogeneity across different cancer types. Such investigations may further expand the therapeutic applicability of targeting the SE complex in oncology.

MED8 is part of the mediator holoenzyme complex that associates with the carboxy-terminal domain of RNA polymerase II, suggesting it may function as a coupling factor that bridges activating or repressive transcription complexes to the RNA Pol II transcriptional machinery [36]. In the present work, we identified that MED8 interacts with CDK7 to assemble SE complexes, and importantly, glioma patients co-expressing high levels of CDK7 and MED8 have an unfavorable prognosis. Thus, pathological evaluation of CDK7 and MED8 serves as a promising prognostic biomarker for stratifying patient survival in glioma. CDK7 is a widely expressed cyclin-dependent kinase-activating kinase that plays a crucial role in activating other CDKs associated with cell-cycle regulation and serves as an essential component of the TFIIH, which phosphorylates the major subunit of RNA Pol II [37]. Moreover, CDK7-mediated phosphorylation of MED1 has been established as a critical mechanism for oncogenic transcription amplification in prostate cancer [38]. However, the regulatory influence of CDK7 on other mediator subunits, including MED8, remained largely unknown. Our current work demonstrates that CDK7 interacts with MED8 to epigenetically activate the PDGFRA oncogene. This finding reveals a previously unrecognized transcriptional dependency in a subset of gliomas and identifies MED8 inhibition as a viable therapeutic approach for epigenetic targeting of PDGFRA-driven gliomas. While our study establishes MED8 as a critical transcriptional regulator in PDGFRA-driven glioma, analysis of public single-cell RNA-seq datasets revealed that the super-enhancer complex score, which includes MED8, is significantly elevated in malignant cells compared to non-malignant cell types within the tumor microenvironment, suggesting that MED8-driven transcriptional programs are a general feature of the malignant compartment. Nevertheless, whether MED8 dependency varies across distinct glioma cellular states—such as the AC-like, MES-like, OPC-like, and NPC-like subtypes [39]—remains to be determined in future studies.

PDGFRA, a member of the receptor tyrosine kinase family, serves as a critical receptor for platelet-derived growth factors (PDGF) [40]. Activation of the PDGF/PDGFRA axis triggers downstream signaling pathways, including the PI3K/AKT/mTOR, RAS/MAPK/ERK, and JAK/STAT3 pathways, which promote cancer cell proliferation, survival, and invasion [41, 42]. In this study, we identified the PDGFRA/ERK signaling cascade as a key pathway regulated by MED8. Notably, our data reveal that pharmacological inhibition of the ERK pathway synergizes with MED8 suppression, further impairing glioma cell viability. Therefore, intervention in the MED8/PDGFRA/ERK axis represents a promising therapeutic strategy for glioma, providing a rational combination approach to enhance treatment efficacy and overcome compensatory resistance mechanisms. Using a genetic approach, we found that disruption of the PDGFRA SE inhibited the transcriptional activity of PDGFRA and glioma cellular proliferation. This approach fundamentally reduces the transcriptional output of the PDGFRA gene, thereby depleting the pool of oncoprotein available for signaling reactivation. Furthermore, cancer cells exhibit ‘transcriptional addiction’ to SE-driven oncogenes like PDGFRA, rendering them uniquely vulnerable to SE disruption, suggesting a potentially favorable therapeutic index. These findings indicated that disrupting the PDGFRA transcriptional activity mediated by the SE complex represents a therapeutic vulnerability in glioma. Further studies are needed to explore whether synergistic inhibition of glioma progression can be achieved through epigenetic and genetic targeting of PDGFRA.

Recent clinical evidence establishes avapritinib as a potent, brain-penetrant PDGFRA tyrosine kinase inhibitor that exhibits significant antitumor activity in preclinical models of glioma with PDGFRA genetic alterations and confers clinical benefit in a subset of patients [4]. However, its efficacy is constrained by several key limitations. As a kinase inhibitor, avapritinib acts post-translationally and does not suppress the high transcriptional output of the PDGFRA oncogene. These insights underscore the necessity of combinatorial strategies that co-target PDGFRA transcription and signaling to achieve durable disease control. Combination therapies have emerged as a promising strategy for overcoming resistance, offering enhanced anti-cancer efficacy and reduced side effects [43, 44]. Building on this, our study introduces a novel glioma therapeutic paradigm co-targeting genetically driven kinase signals and epigenetic drivers. Repurposing venetoclax as a MED8-targeting agent, we directly target epigenetics to suppress PDGFRA transcriptional hyperactivation—the root of its overexpression. Combined with avapritinib’s PDGFRA kinase inhibition, this exerts dual-layer effects: disrupting oncogene “production” (transcriptional) and blocking “function” (post-translational). Beyond mere drug combination, this synergistic co-targeting of two interdependent cancer hallmarks provides a comprehensive, mechanism-based strategy to overcome glioma therapeutic resistance. Notably, venetoclax is a cornerstone of hematologic malignancy therapy with a proven clinical safety profile [45, 46]. The significant anti-tumor efficacy of venetoclax in our orthotopic model provides functional evidence that it reaches therapeutically relevant concentrations at the intracranial tumor site. This finding is consistent with prior preclinical studies demonstrating venetoclax activity in other CNS malignancies and its favorable physicochemical properties for blood-brain barrier penetration [24, 47, 48]. However, we acknowledge that direct pharmacokinetic measurement of brain tumor drug concentrations in PDGFRA-driven glioma models represents an important future direction to optimize dosing strategies for clinical translation. While we utilized PDCs, PDC xenograft and PDOs to enhance clinical relevance, validation in patient-derived xenograft (PDX) models was not performed. Future studies incorporating PDX models will be essential to further establish the translatability of the venetoclax-avapritinib combination for PDGFRA-driven glioma.

Given that the combination of venetoclax and avapritinib has not been previously explored in glioma, we conducted the first preclinical evaluation of its efficacy. Our results demonstrate that the dual inhibition strategy produces a synergistic antitumor effect, surpassing the efficacy of either agent alone at equivalent doses. Furthermore, our findings support molecular alteration-based patient stratification for glioma. Patients with PDGFRA alterations but without MED8-mediated epigenetic activation may respond to avapritinib monotherapy [4]. Conversely, tumors with MED8-driven PDGFRA transcriptional activation but no PDGFRA alterations benefit from venetoclax. Notably, the venetoclax-avapritinib combination co-targets dual oncogenic layers in patients with both alterations, boosting efficacy and overcoming resistance. Preclinically validated, this stratified precision therapy requires clinical validation in well-designed trials.

## Conclusions

This study is the first to comprehensively identify a key component of the SE complex in glioma, demonstrating MED8 as a key driver of transcription for SE-associated oncogenes. This finding offers novel insights into MED8’s role in mediating the epigenetic activation of PDGFRA. Furthermore, we repurposed venetoclax as the first-in-class MED8-targeting agent, which exerts potent synergy with avapritinib to effectively suppress PDGFRA-driven glioma growth in preclinical models. This combinatorial strategy, co-targeting the dual genetic-epigenetic regulatory network, constitutes a robustly clinically translatable therapeutic modality for PDGFRA-driven glioma. 

## Supplementary Information

Supplementary Material 1.

## Data Availability

The data analyzed during this study are included in this published article and the supplemental data files. Additional supporting data are available from the corresponding authors upon reasonable request.

## Abbreviations

PDGFRA: Platelet-derived growth factor receptor alpha
SE: Super-enhancer
RNA Pol II: RNA polymerase II
GSCs: Glioma stem cells
PDCs: Patient-derived cells
PDOs: Patient-derived organoids
IF: Immunofluorescence
IHC: Immunohistochemistry
RT-qPCR: Reverse transcription quantitative PCR
CRISPRi: CRISPR interference
Co-IP: Co-immunoprecipitation
ChIP: Chromatin immunoprecipitation
CETSA: Cellular thermal shift assay
IC50: Half-maximal inhibitory concentration
NHA: Normal human astrocytes
TFIIH: Transcription factor IIH
Rluc-PCA: Renilla luciferase protein fragment complementation assay
