# Supplementary material for: Disrupting MED8-dependent epigenetic reprogramming augments avapritinib sensitivity in PDGFRA-driven glioma
Source: J Exp Clin Cancer Res. 2026 May 19;45:159. doi: 10.1186/s13046-026-03736-0 (PMC13360484; doi:10.1186/s13046-026-03736-0)
Supplement: Supplementary file 1 — Supplementary Material 1. [file 13046_2026_3736_MOESM1_ESM.docx]

**Supplementary Tables**

**Supplementary Table S1**

| Characteristic | n of patients |
| --- | --- |
|  |  |
| KPS |  |
| ≥80 | 25 |
| <80 | 5 |
| Gender |  |
| Male | 18 |
| Female | 12 |
| Age |  |
| ≤ 50 | 11 |
| > 50 | 19 |
| Grade |  |
| LGG | 12 |
| GBM | 18 |

**Supplementary Table S2**

| shRNA name | Sequence | |
| --- | --- | --- |
| sh-MED8 | GAAGCAACUGACGACAGAU | AUCUGUCGUCAGUUGCUUC |
| sh-CDK7 | CAACAUUGGAUCCUACAUA | UAUGUAGGAUCCAAUGUUG |
| sh-BCL2 | AACAUCGCCCUGUGGAUGACU | AGUCAUCCACAGGGCGAUGUU |

**Supplementary Table S3**

| Antibodies | Source | Reactivity | Manufacturer | Catalog | Application dilution |
| --- | --- | --- | --- | --- | --- |
| MED8 | Mouse | Human, Mouse | Santa Cruz | **sc-365713** | WB, 1:1000/ IP, 1:200/ IF, 1:500 |
| β-actin | Mouse | Human, Mouse | proteintech | 66009-1-Ig | WB, 1:20000 |
| Caspase-3 | Rabbit | Human, Mouse | Cell Signaling | 9662 | WB, 1:1000 |
| Cleaved Caspase-3 | Rabbit | Human, Mouse | Cell Signaling | **9661** | WB, 1:1000 |
| Cyclin B1 | Rabbit | Human, Mouse | proteintech | 55004-1-AP | WB, 1:1000 |
| Ki67 | Rabbit | Human | proteintech | 27309-1-AP | IHC, 1:2000/ IF, 1:500 |
| γ-H2AX | Rabbit | Human, Mouse | proteintech | 10856-1-AP | IHC, 1:500/ IF, 1:1000 |
| ERK | Rabbit | Human, Mouse | proteintech | **11257-1-AP** | WB, 1:2000 |
| p-ERK | Rabbit | Human, Mouse | proteintech | 28733-1-AP | WB, 1:1000 |
| p-P38 | Rabbit | Human, Mouse | proteintech | **28796-1-AP** | WB, 1:1000 |
| p-JNK | Rabbit | Human, Mouse | proteintech | **80024-1-RR** | WB, 1:1000 |
| PDGFRA | Rabbit | Human, Mouse | Cell Signaling | **3174** | WB, 1:1000/ IF, 1:500 |
| KDR | Rabbit | Human, Mouse | proteintech | **26415-1-AP** | WB, 1:1000 |
| CDK7 | Mouse | Human, Mouse | Cell Signaling | 2916 | WB, 1:2000/ IP, 1:200 |
| CDK7 | Mouse | Human, Mouse | proteintech | 67889-1-Ig | WB, 1:1000/ IF, 1:750 |
| HA | Rabbit | Human, Mouse | Cell Signaling | **3724** | WB, 1:1000/ IP, 1:50 |
| H3K27ac | Rabbit | Human, Mouse | Cell Signaling | **8173** | WB, 1:1000/ ChIP, CUT&Tag, 1:50 |
| RNA pol II | Mouse | Human, Mouse | Cell Signaling | **2629** | WB, 1:1000/ ChIP, 1:50 |
| BCL2 | Rabbit | Human | proteintech | **12789-1-AP** | WB, 1:2000 |

**Supplementary Table S4**

| Primers | Forward | | Reverse |
| --- | --- | --- | --- |
| MED8 | CAGGGCAGACTGGTAGCAAA | | GCGTCACTTTTTGTGGGGAC |
| GAPDH | AGCAAGAGCACAAGAGGAAG | | GGTTGAGCACAGGGTACTTT |
| EPHA2 | GAGAAGGATGGCGAGTTCAG | | AGGTTGCTGTTGACGAGGAT |
| PRKACA | AAATGCGCAGCCTGACAGAT | | CACAGGGTGAGAACGAAGCA |
| CACNG4 | CAATGACTACGACCACGACAG | | GCAGCCACGAAGAGGATG |
| RAP1B | GAGGAGGTTGTGGTGGATGA | | CTCCATTAACTGCCGAATGAT |
| TGFB2 | GCAAAGTTGTGAAAACAAGAGC | | ATCCCAGGTTCCTGTCTTTATG |
| TGFBR1 | GTAGCTCTGATGAGTGCAATGAC | | CAGATATGGCAACTCCCAGTG |
| MAP3K4 | AGGATGATTCTCTTGGCTGG | | TCGGGCTTCTCCAAGTC |
| KDR | GGAGCTTAAGAATGCATCCTTG | | GATGCTTTCCCCAATACTTGTC |
| PDGFRA | ATTGTGGAGAATCTGCTGCCTGGA | | GAAGCTGTCTTCCACCAGGTCTGA |
| TGFA | GGTCCGAAAACACTGTGAGTGG | | CAAACTCCTCCTCTGGGCTCTT |
| Primers | | Forward | Reverse |
| PDGFRA-Pro | | CCGCTCGCATTACTTCCAC | TTTGTTCCCGCTCATTTTCT |
| PDGFRA-E1 | | CAAGGCAGTCTCCTTTCCAG | ACAATGGGGACTGCAGAATC |
| PDGFRA-E2 | | AGCCATGTTTTCCGACTCTG | GGCGTCAAATTCAGCTTTTC |
| PDGFRA-E3 | | AAGCCAAAAATCACCTGTCC | GTCCAGCCCAATTTGTGTTT |
| PDGFRA-E4 | | GGGGACGTGTCTTAGTGCAT | ACTTGGACTCCCCAGTCTCC |
| PDGFRA-E5 | | GAAGGGGCAAACTCTGTGTC | AGAGAGCTTCCTGGCCTTTC |

**Supplementary Table S5**

| Name | sgRNA-E1 |
| --- | --- |
| RefSeq# | ACAAATGCTGTGCCATCACA |
| Name | sgRNA-E2 |
| RefSeq# | ATGAACGCTATGTTCTTACC |

**Supplementary Table S6**

|  | **ligand_name** | **scores** | **MW (g/mol)** | **XLogP3-AA** |
| --- | --- | --- | --- | --- |
| 1 | Dalbavancin | -9.5 | 1816.7 | 3.8 |
| 2 | Daptomycin | -9.5 | 1620.7 | -5.1 |
| 3 | Venetoclax | -9.4 | 868.4 | 8.2 |
| 4 | Rezafungin | -8.7 | 1226.4 | 2.9 |
| 5 | Anidulafungin | -8.5 | 1140.2 | 2.4 |
| 6 | Cyclosporin A | -8.2 | 1202.6 | 7.5 |
| 7 | Abarelix | -8.1 | 1416.1 | 3.7 |
| 8 | Roxithromycin | -8.0 | 837.0 | 3.1 |
| 9 | Tannic acid | -7.8 | 1701.2 | 6.2 |
| 10 | Vincristine | -7.8 | 825.0 | 2.8 |

XLogP3-AA: Liposolubility of the molecule. The data is sourced from PubChem.

**Supplementary Table S7**

| id | Category | id | Category |
| --- | --- | --- | --- |
| MED8 | Co-activator/repressors | MED10 | Co-activator/repressors |
| MED31 | Co-activator/repressors | MED27 | Co-activator/repressors |
| CDK8 | Helix-turn-helix | MED13 | Co-activator/repressors |
| MED1 | Helix-turn-helix | MED15 | Co-activator/repressors |
| MED28 | Co-activator/repressors | HDAC1 | Histones Modify Enzymes |
| MED26 | Co-activator/repressors | MED11 | Co-activator/repressors |
| POLR2E | Other Cofactors | BRD4 | Chromatin Remodeling Factors |
| POLR2H | Other Cofactors | YY1 | Other Cofactors |
| HDAC2 | Histones Modify Enzymes | POLR2G | Other Cofactors |
| SOX2 | Helix-turn-helix | MED22 | Co-activator/repressors |
| CDK12 | Cell Cycle | MED7 | Co-activator/repressors |
| POLR2F | Other Cofactors | CHD7 | Chromatin Remodeling Factors |
| EP300 | Other Cofactors | MED23 | Co-activator/repressors |
| MED16 | Co-activator/repressors | CDK9 | Cell Cycle |
| SMC1A | Other Cofactors | MED18 | Co-activator/repressors |
| SMARCB1 | Chromatin Remodeling Factors | MED12L | Co-activator/repressors |
| POLR2A | General Cofactors | MED12 | Co-activator/repressors |
| KDM5C | Histones Modify Enzymes | SMARCA4 | Chromatin Remodeling Factors |
| MED21 | Co-activator/repressors | MBD3 | Helix-turn-helix |
| POLR2J | Other Cofactors | MED6 | Co-activator/repressors |
| POLR2L | Other Cofactors | MED30 | Co-activator/repressors |
| MED13L | Co-activator/repressors | MED20 | Co-activator/repressors |
| MED29 | Co-activator/repressors | POLR2M | Other Cofactors |
| CREBBP | Co-activator/repressors | MED9 | Co-activator/repressors |
| MED14 | Co-activator/repressors | FOXO3 | Helix-turn-helix |
| MED4 | Co-activator/repressors | POLR2D | Other Cofactors |
| MED19 | Co-activator/repressors | CHD4 | Chromatin Remodeling Factors |
| POLR2K | Other Cofactors | KDM6A | Histones Modify Enzymes |
| KDM1A | Histones Modify Enzymes | CCNC | Cell Cycle |
| MED25 | Co-activator/repressors | MED24 | Co-activator/repressors |
| POLR2B | Other Cofactors | POLR2C | Other Cofactors |
| MED17 | Co-activator/repressors | NIPBL | Other Cofactors |
| CDK7 | Cell Cycle |  |  |

**Supplementary S8**

| id | coef | HR | HR.95L | HR.95H | pvalue |
| --- | --- | --- | --- | --- | --- |
| MED8 | 1.006555 | 2.736158 | 1.77729 | 4.212347 | 4.82E-06 |
| MED14 | 0.292595 | 1.3399 | 0.992447 | 1.808997 | 0.056074 |
| CDK7 | 0.671167 | 1.956519 | 1.174179 | 3.260123 | 0.009986 |
| MED10 | 0.448069 | 1.565286 | 1.026909 | 2.385917 | 0.037212 |
| HDAC1 | 0.50911 | 1.66381 | 1.248772 | 2.216787 | 0.000506 |
| MED30 | -0.29175 | 0.746952 | 0.54334 | 1.026865 | 0.072383 |

**Supplementary Figures**

**Supplementary Fig. S1**

**
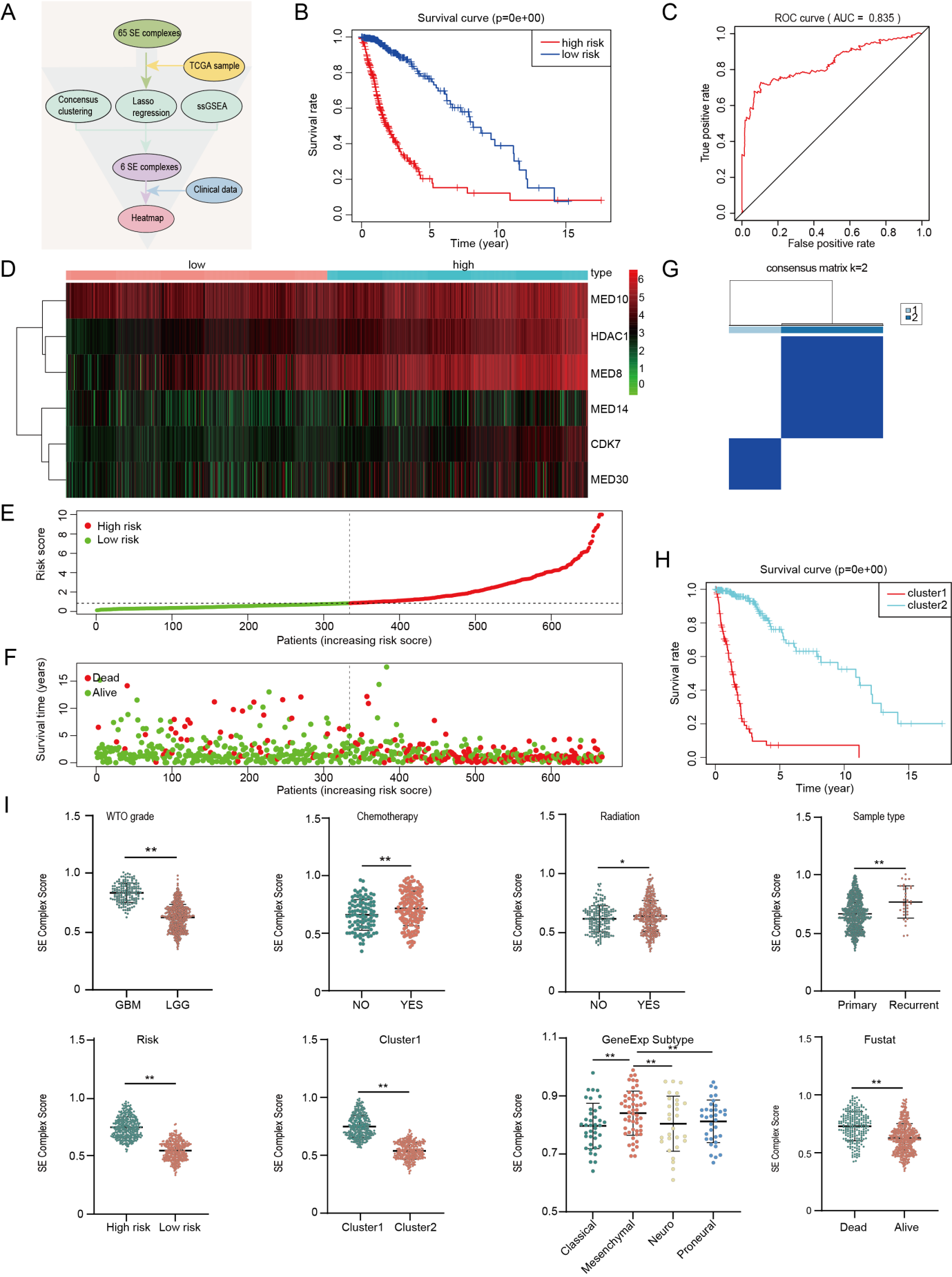
Supplementary Fig. S1 Three clustering methods divide patients into two groups.** (A) Flow chart of genetic screening. (B) Kaplan-Meier survival curves of two risk groups. (C) The ROC curve indicates the sensitivity and specificity of predicting the overall survival of patients. (D) The expression profile of 6 SE complex components in two risk groups. (E-F) Distribution of survival status, overall survival, and risk score. (G) The consensus score matrix of all samples when k = 2. (H) Kaplan-Meier survival curves showed differences in overall survival among two subclusters. (I) Differences in SE complex scores among different clinical pathological features. Data represent three independent biological replicates, each performed with technical duplicates or triplicates. Data are presented as mean ± SD. *, p < 0.05; **, p < 0.01; ns, not significant.

**Supplementary Fig. S2**

**
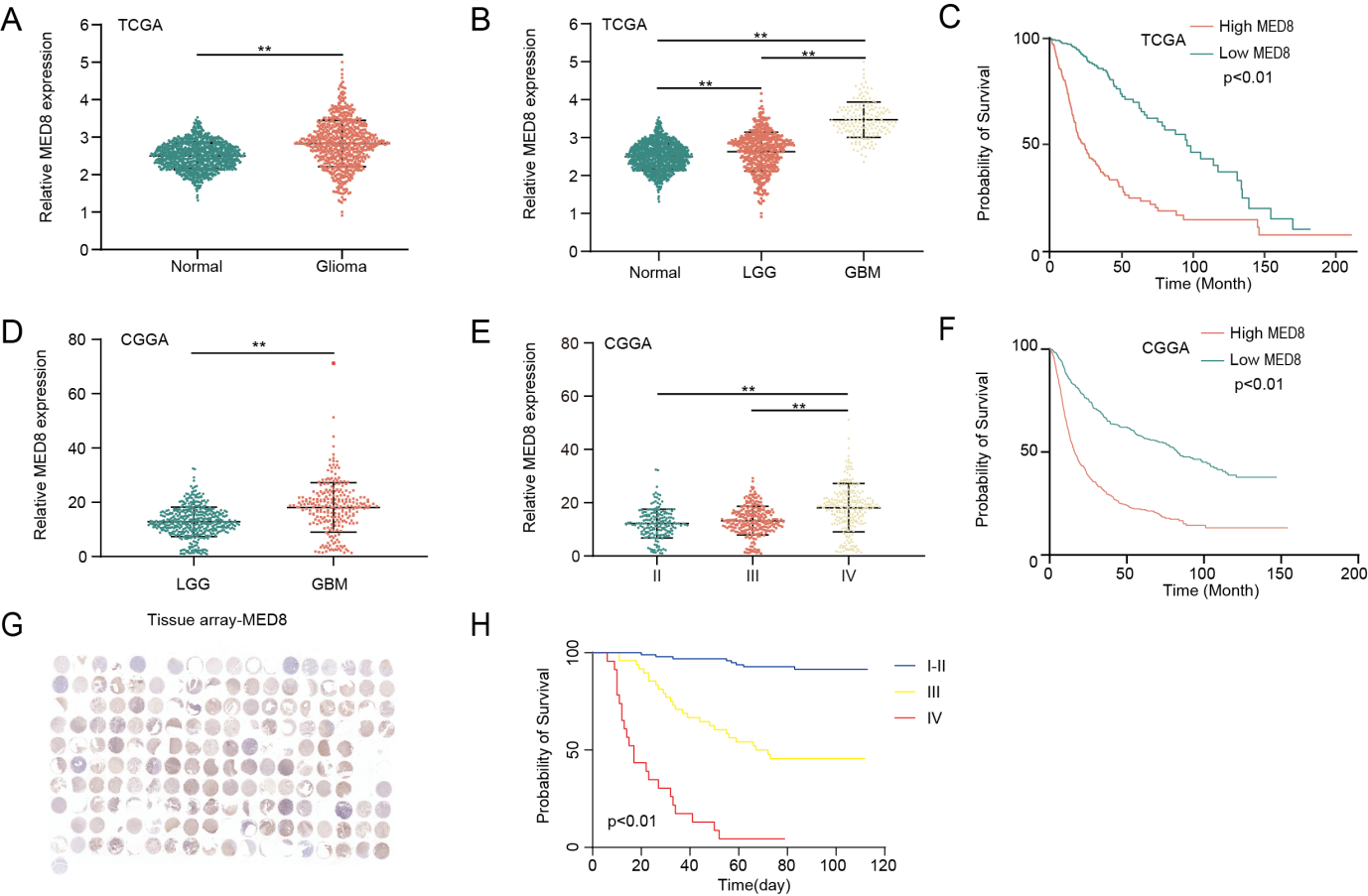
**

**Supplementary Fig. S2 Expression of SE complex MED8 in glioma.** (A-F) Expression level and Kaplan-Meier survival analysis of MED8 in the TCGA and CGGA databases. (G) Overall staining of MED8 in the tissue microarray. The microarray contained 180 samples and one control spot. (H) Kaplan-Meier survival analysis on data from the tissue microarray according to tumor grades. Data represent three independent biological replicates, each performed with technical duplicates or triplicates. Data are presented as mean ± SD. *, p < 0.05; **, p < 0.01; ns, not significant.

**Supplementary Fig. S3**

**
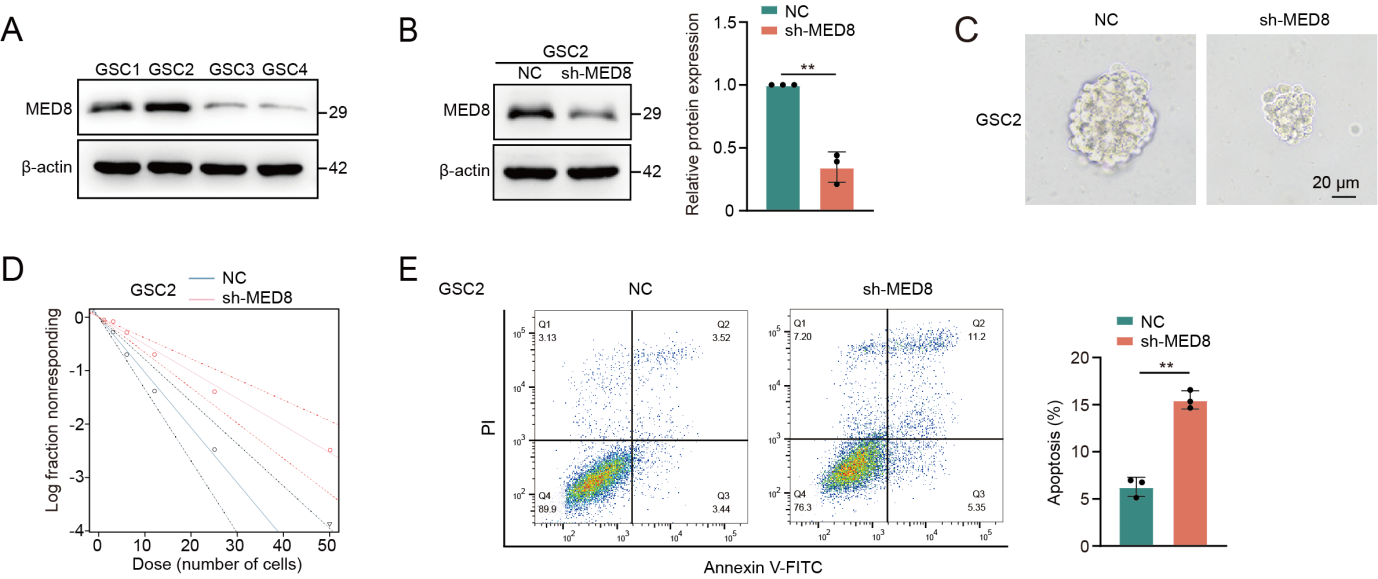
**

**Supplementary Fig. S3 MED8 promotes glioma progression in GSCs.** (A) Western blot analysis of MED8 in four GSC lines. (B) Western blot analysis of MED8 in NC/sh-MED8 GSC2. (C-D) Tumor spheres and limiting dilution assays were performed for GSC2 transfected with sh-NC or sh-MED8. (E) Stained with PI and annexin V-FITC for apoptotic analysis in NC/sh-MED8 GSC2. Data represent three independent biological replicates, each performed with technical duplicates or triplicates. Data are presented as mean ± SD. *, p < 0.05; **, p < 0.01; ns, not significant.

**Supplementary Fig. S4**

**
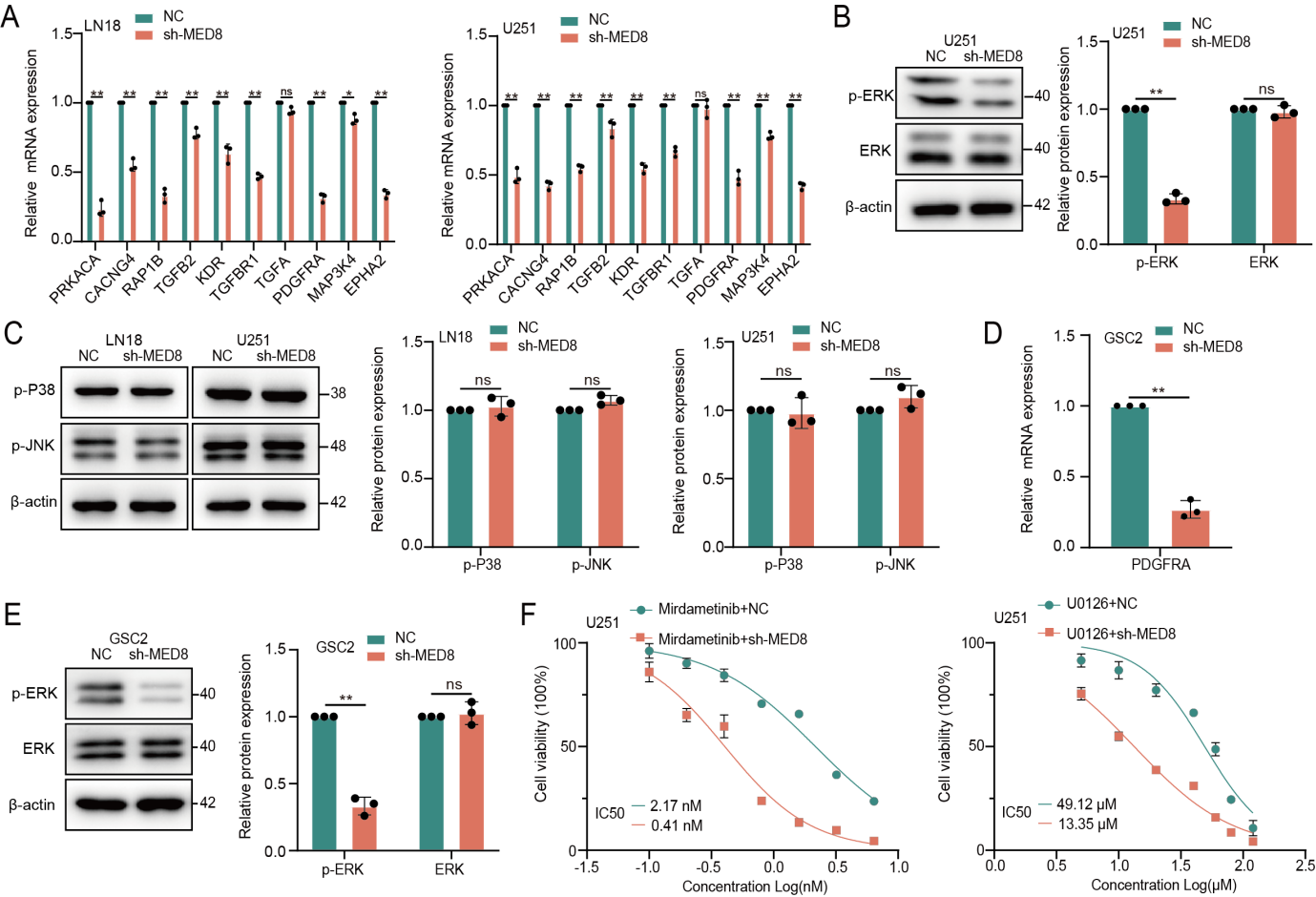
**

**Supplementary Fig. S4 MED8 regulates MAPK signaling pathway.** (A) RT-qPCR analysis of the top ten differential genes in the MAPK signaling pathway in NC/sh-MED8 LN18 and U251 cells. (B) Western blot analysis of p-ERK and ERK in NC/sh-MED8 U251 cells. (C) Western blot analysis of p-P38 and p-JNK in NC/sh-MED8 LN18 and U251 cells. (D) RT-qPCR analysis of PDGFRA in NC/sh-MED8 GSC2. (E) Western blot analysis of p-ERK and ERK in NC/sh-MED8 GSC2. (F) CCK8 assays were performed to examine the effect of MED8 knockdown on the IC50 of ERK kinase inhibitors (U0126 and mirdametinib) in U251 cells. Data represent three independent biological replicates, each performed with technical duplicates or triplicates. Data are presented as mean ± SD. *, p < 0.05; **, p < 0.01; ns, not significant.

**Supplementary Fig. S5**

**
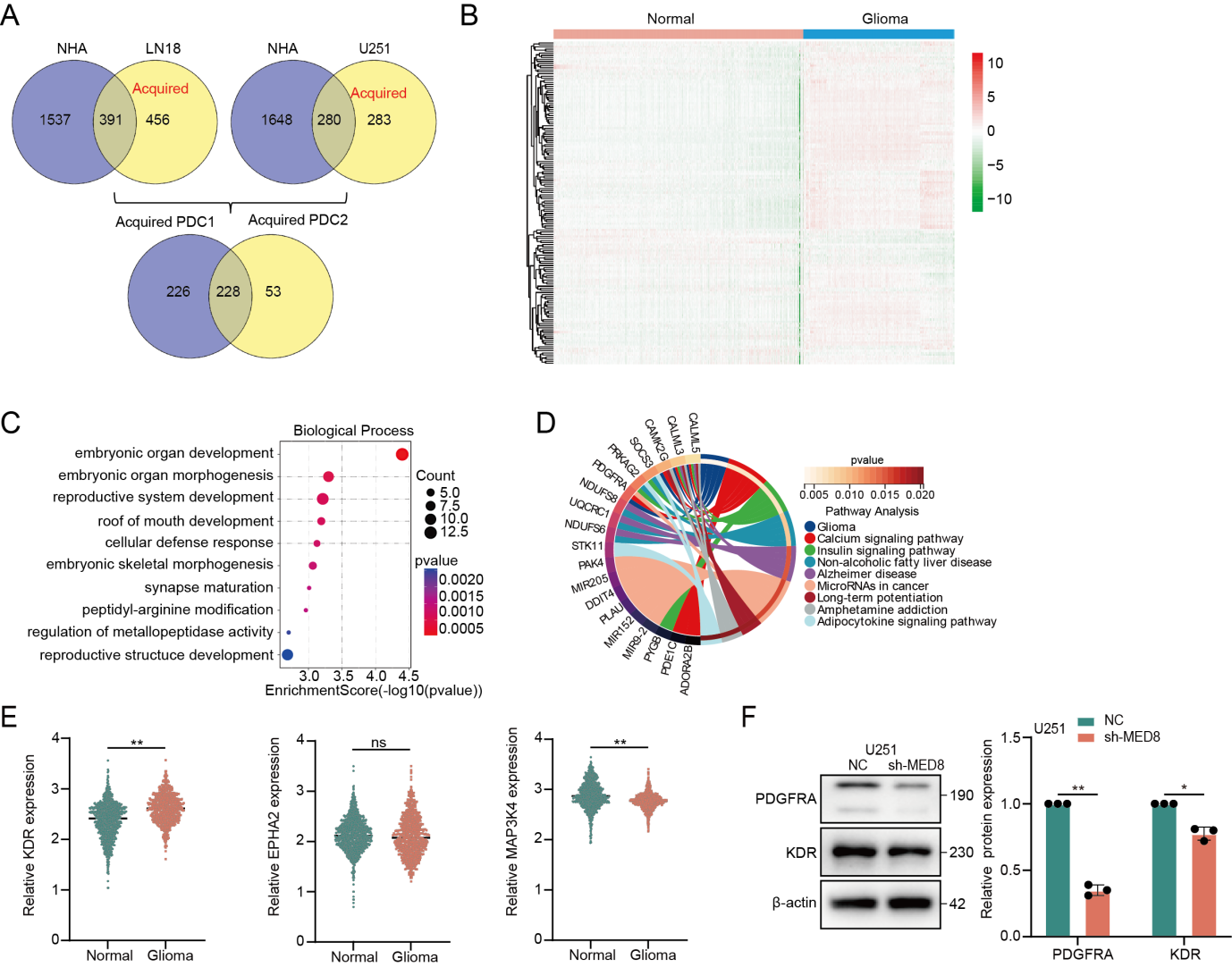
**

**Supplementary Fig. S5 PDGFRA is a crucial target of MED8-mediated SE.** (A) SE-associated genes in LN18 and U251 cells were systematically compared with the SE-associated genes in NHA cells. 228 glioma-SE genes were commonly acquired in LN18 and U251 cells. (B) The expression profile of the glioma-SE genes was clearly segregated between normal and glioma samples in the unsupervised clustering analysis. (C-D) The GO and KEGG analysis of the glioma-SE genes. (E) Expression level of KDR, EPHA2, and MAP3K4 in the TCGA database. (F) Western blot analysis of PDGFRA and KDR in NC/sh-MED8 U251 cells. Data represent three independent biological replicates, each performed with technical duplicates or triplicates. Data are presented as mean ± SD. *, p < 0.05; **, p < 0.01; ns, not significant.

**Supplementary Fig. S6**

**
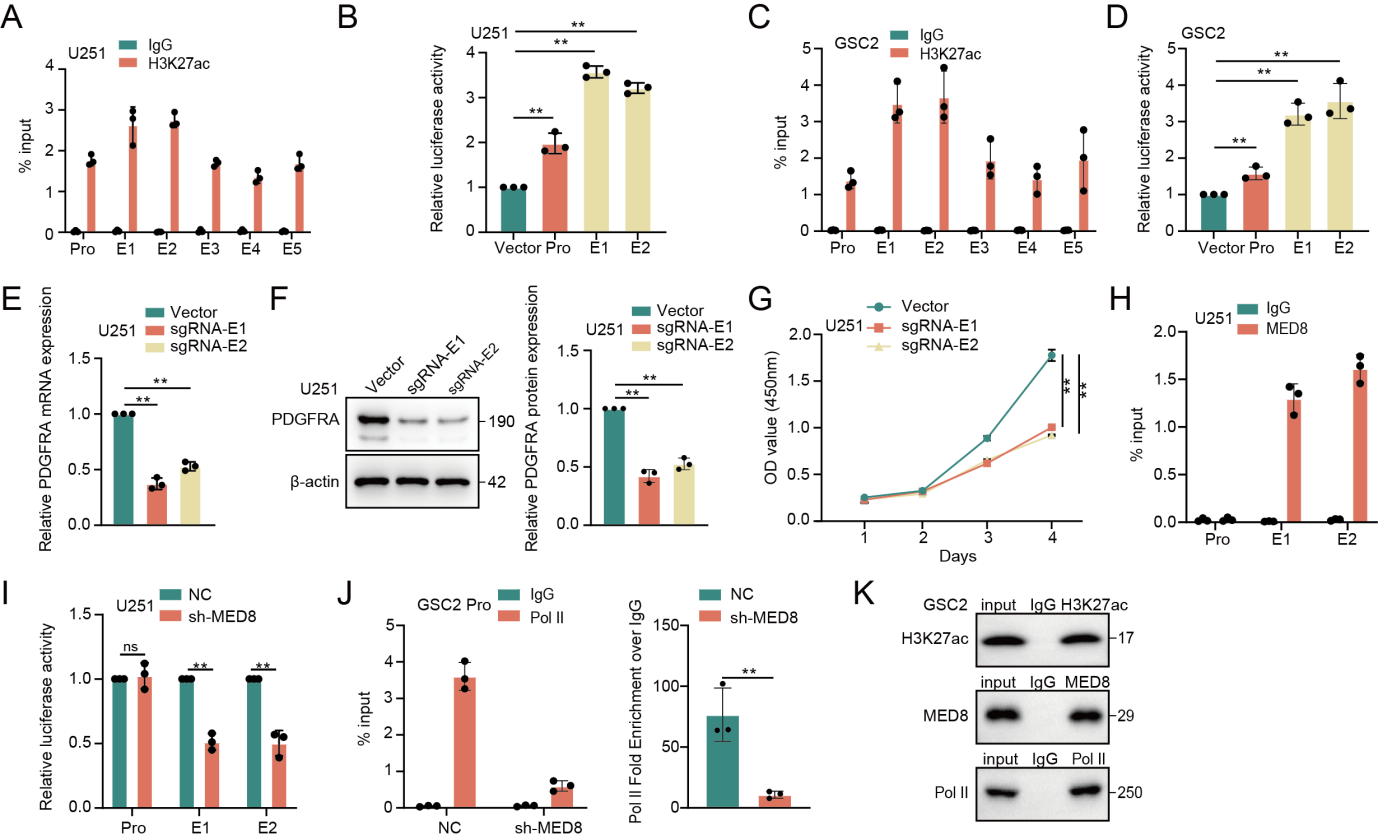
**

**Supplementary Fig. S6. MED8 regulates the SE of PDGFRA.** (A) ChIP-qPCR was performed to quantify the enrichment of H3K27ac at the PDGFRA promoter and enhancer regions in U251 cells. (B) Luciferase reporter assays measured promoter and enhancer activity of PDGFRA in U251 cells. (C) ChIP-qPCR was performed to quantify the enrichment of H3K27ac at the PDGFRA promoter and enhancer regions in GSC2. (D) Luciferase reporter assays measured promoter and enhancer activity of PDGFRA in GSC2. (E-F) Blockade of PDGFRA SE critical regions by sgRNA reduced the expression of PDGFRA in U251 cells. (G) Blockade of PDGFRA SE critical regions by sgRNA reduced the proliferation of U251 cells. (H) The enrichment of MED8 on the promoter and enhancer of PDGFRA was analyzed with ChIP assays in U251 cells. (I) Luciferase reporter assays measured promoter and enhancer activity of PDGFRA in NC/sh-MED8 U251 cells. (J) ChIP-qPCR was performed to quantify the enrichment of Pol II at the PDGFRA promoter region in NC/sh-MED8 GSC2. (K) ChIP samples from H3K27ac/MED8/Pol II antibody or control IgG were immunoblotted with anti-H3K27ac/MED8/Pol II antibody. Input served as a loading control. Data represent three independent biological replicates, each performed with technical duplicates or triplicates. Data are presented as mean ± SD. *, p < 0.05; **, p < 0.01; ns, not significant.

**Supplementary Fig. S7**

**
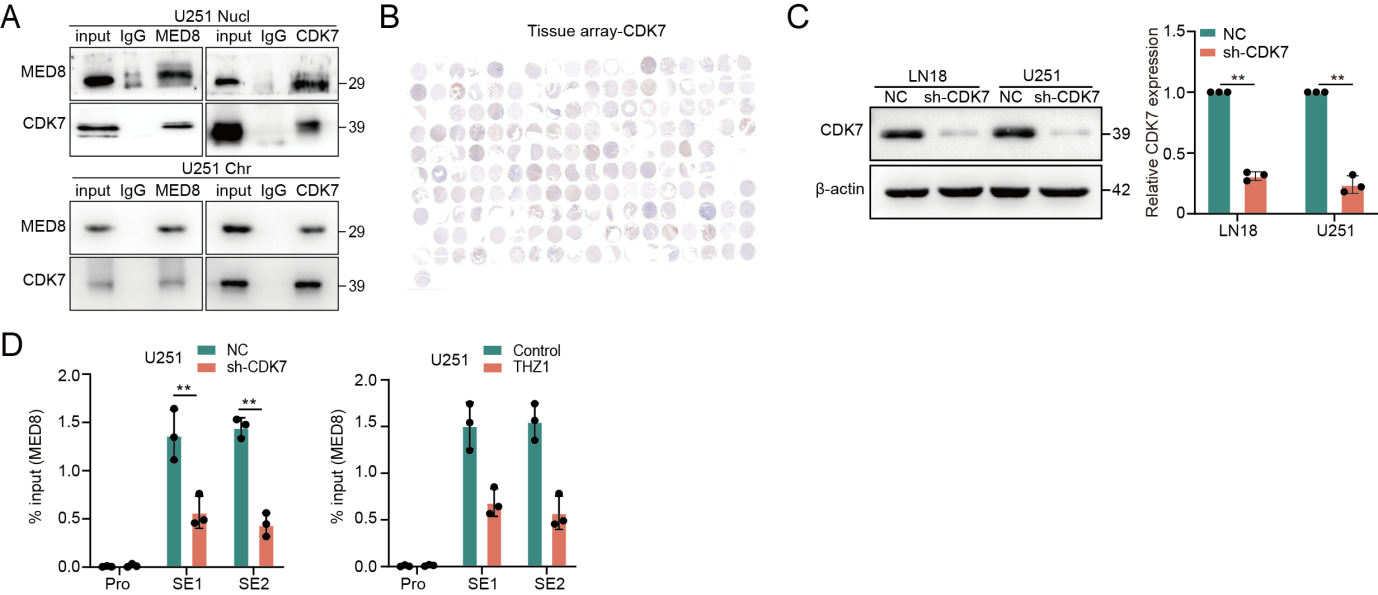
**

**Supplementary Fig. S7. MED8 interacts with CDK7 to regulate the SE of PDGFRA.** (A) Reciprocal Co-IP analysis demonstrated the interaction between MED8 and CDK7 in the U251 nucleus and chromatin. (B) Overall staining of MED8 in the tissue microarray. (C) Western blot analysis of CDK7 in NC/sh-CDK7 LN18 and U251 cells. (D) After sh-CDK7 or THZ1 treatment of U251 cells, ChIP assays were performed to analyze the enrichment of MED8 on the promoter and enhancer of PDGFRA. Data represent three independent biological replicates, each performed with technical duplicates or triplicates. Data are presented as mean ± SD. *, p < 0.05; **, p < 0.01; ns, not significant.

**Supplementary Fig. S8**

**
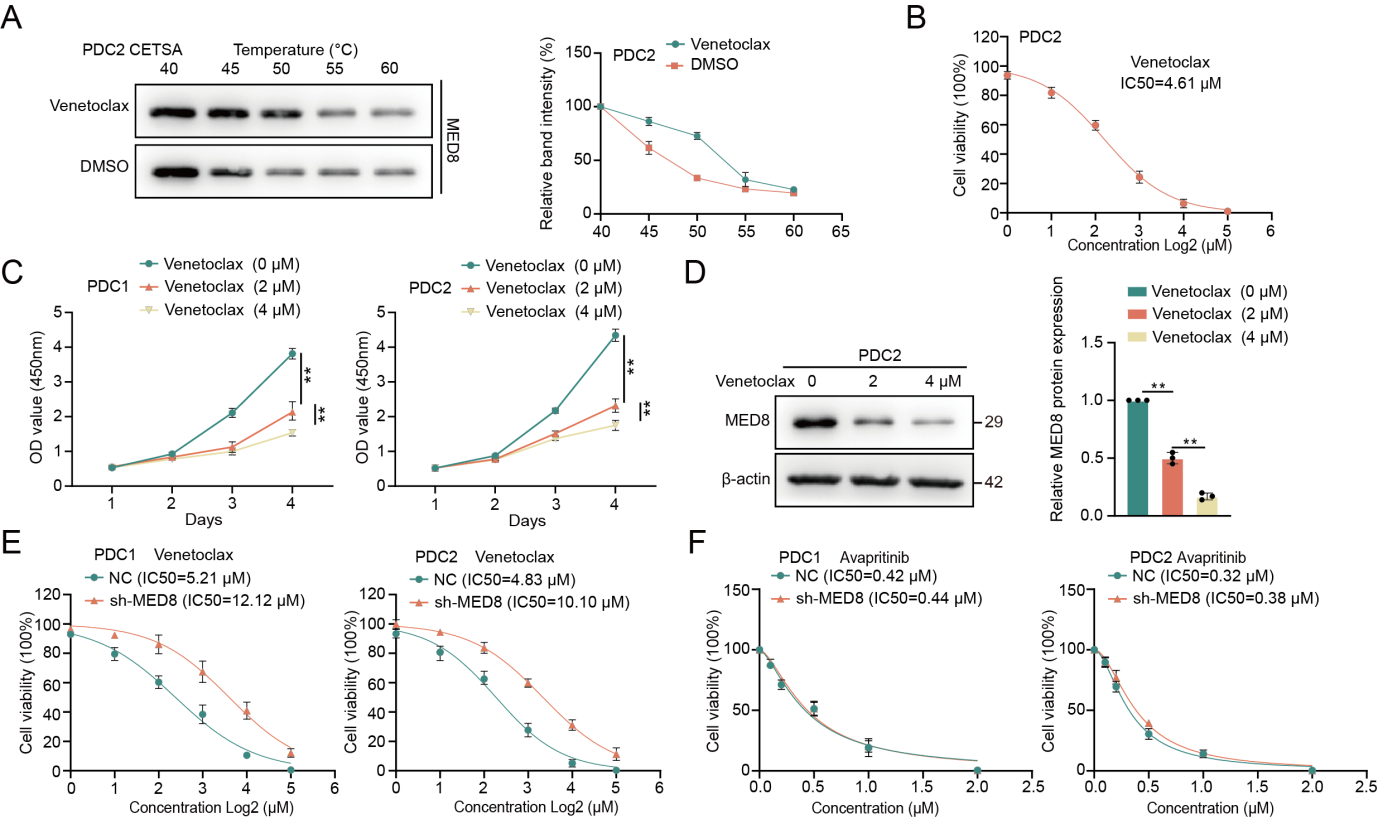
**

**Supplementary Fig. S8. MED8 inhibitor venetoclax inhibits the progression of glioma.** (A) PDC2 were treated with DMSO and venetoclax (4 μM) for 2 h and heated at the indicated temperatures and then lysed for western blot analysis to detect the expression of MED8 protein. CETSA curves indicate relative MED8 band intensity. (B) PDC2 were treated with 1, 2, 4, 8, 16, and 32 μM doses of venetoclax for 48 h. The cell viability was assessed by CCK-8 analysis. (C) Western blot analysis of MED8 in PDC2 treated with 2 and 4 μM doses of venetoclax. (D) CCK8 assays were performed to examine the proliferation in PDCs treated with 2 and 4 μM doses of venetoclax. (E) PDCs were treated with 1, 2, 4, 8, 16, and 32 μM doses of venetoclax for 48 h. The cell viability was assessed by CCK-8 analysis. (F) PDCs were treated with 0.1, 0.2, 0.5, 1.0, and 2.0 μM doses of avapritinib for 48 h. Data represent three independent biological replicates, each performed with technical duplicates or triplicates. Data are presented as mean ± SD. *, p < 0.05; **, p < 0.01; ns, not significant.

**Supplementary Fig. S9**

**
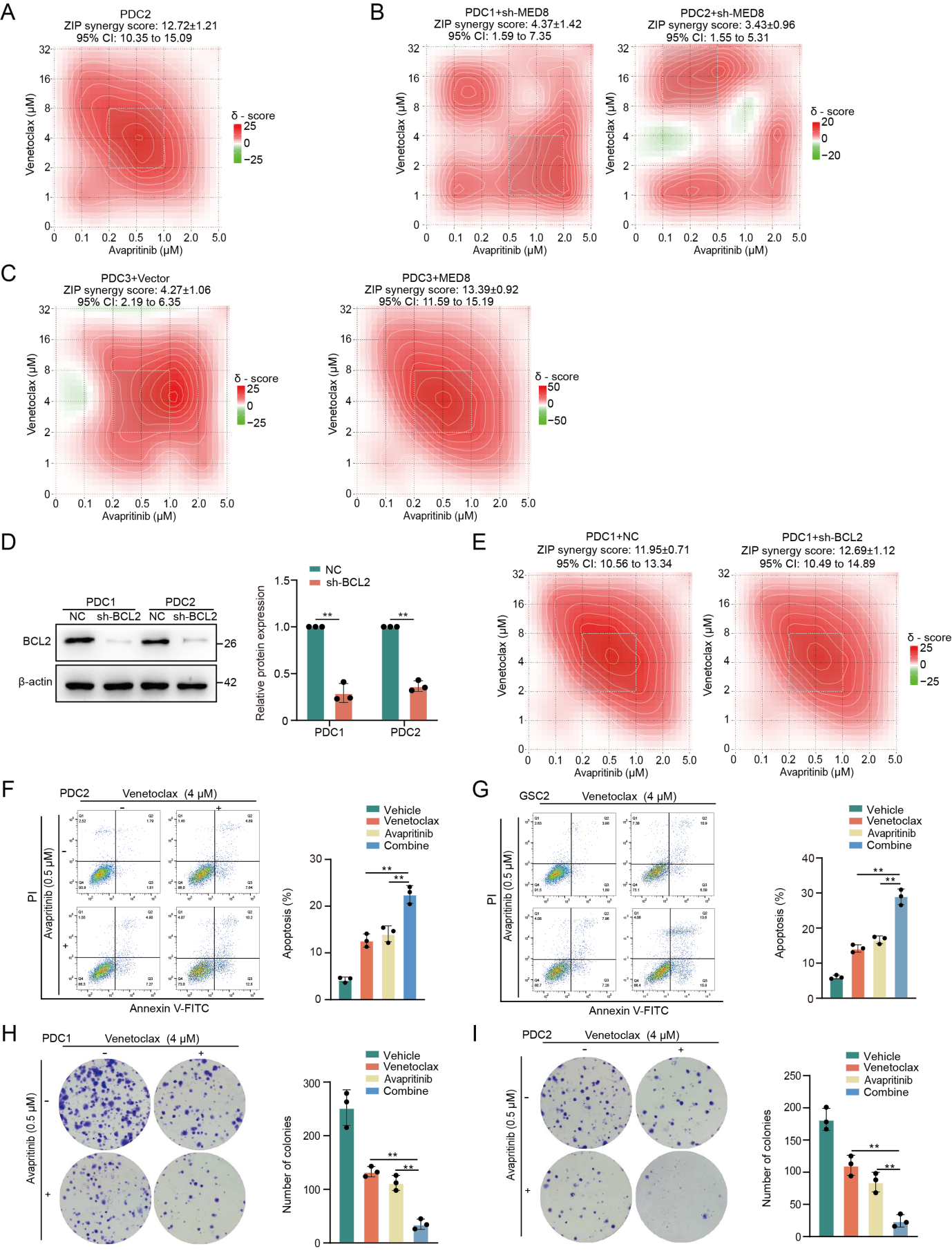
**

**Supplementary Fig. S9 Synergistic effects of MED8 inhibitor venetoclax and avapritinib in vitro.** (A) Synergy efficacy was observed using the combination of venetoclax and avapritinib in PDC2 that was assessed by the ZIP synergy score. (B) Synergy efficacy was observed using the combination of venetoclax and avapritinib in sh-MED8 PDCs. (C) Synergy efficacy was observed using the combination of venetoclax and avapritinib in Vector/MED8 PDC3. (D) Western blot analysis of BCL2 in NC/sh-BCL2 PDCs. (E) Synergy efficacy was observed using the combination of venetoclax and avapritinib in NC/sh-BCL2 PDC1. (F-G) PDC2 and GSC2 were treated with venetoclax and avapritinib for 48 h and harvested for apoptotic analysis. (H-I) Colony formation assays were performed in PDCs treated with venetoclax and avapritinib. Data represent three independent biological replicates, each performed with technical duplicates or triplicates. Data are presented as mean ± SD. *, p < 0.05; **, p < 0.01; ns, not significant.

**Supplementary Fig. S10**


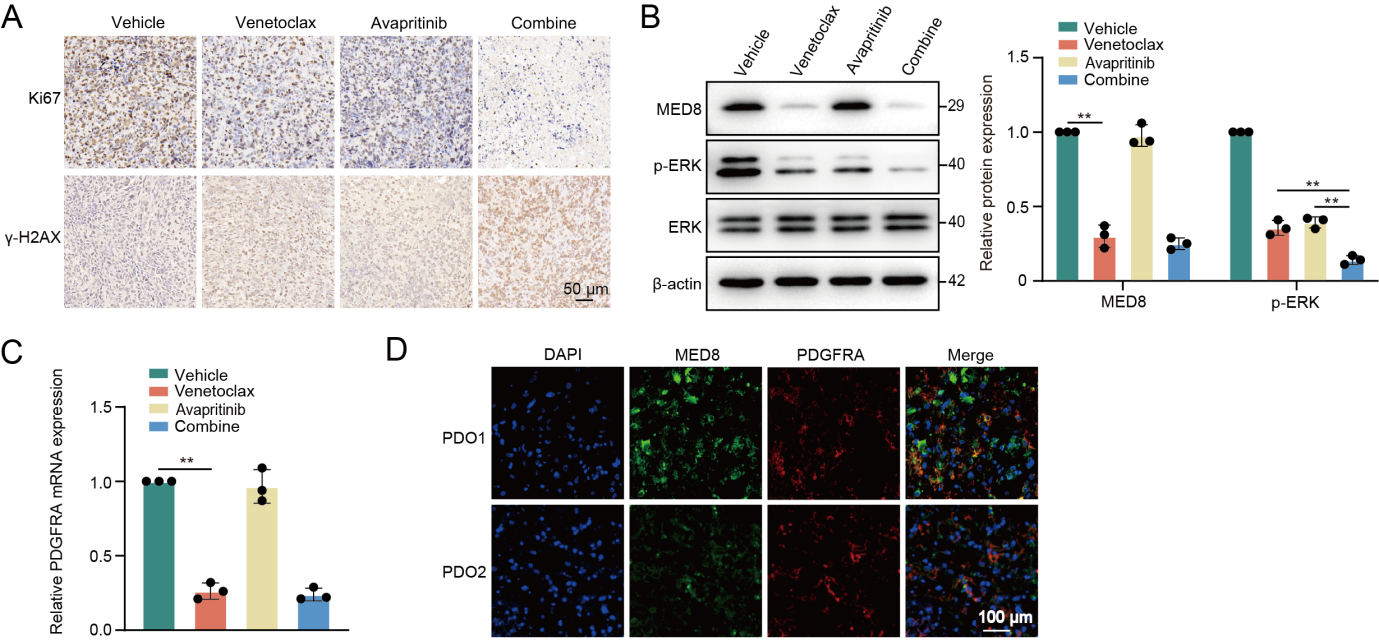


**Supplementary Fig. S10 Synergistic effects of MED8 inhibitor venetoclax and avapritinib in vivo.** (A) Representative Ki-67 and γ-H2AX IHC staining in PDC mice xenografts. (B) Western blot analysis of MED8, p-ERK and ERK in PDC mice xenografts. (C) RT-qPCR analysis of PDGFRA in PDC mice xenografts. (D) IF of MED8 and PDGFRA in PDOs. Data represent three independent biological replicates, each performed with technical duplicates or triplicates. Data are presented as mean ± SD. *, p < 0.05; **, p < 0.01; ns, not significant.
